# Newly Developed Mg^2+^–Selective Fluorescent Probe Enables Visualization of Mg^2+^ Dynamics in Mitochondria

**DOI:** 10.1371/journal.pone.0023684

**Published:** 2011-08-16

**Authors:** Yutaka Shindo, Tomohiko Fujii, Hirokazu Komatsu, Daniel Citterio, Kohji Hotta, Koji Suzuki, Kotaro Oka

**Affiliations:** 1 Center for Biosciences and Informatics, School of Fundamental Science and Technology, Graduate School of Science and Technology, Keio University, Yokohama, Kanagawa, Japan; 2 Center for Science and Technology for Designing Functions, School of Integrated Design Engineering, Graduate School of Science and Technology, Keio University, Yokohama, Kanagawa, Japan; INSERM U901, France

## Abstract

Mg^2+^ plays important roles in numerous cellular functions. Mitochondria take part in intracellular Mg^2+^ regulation and the Mg^2+^ concentration in mitochondria affects the synthesis of ATP. However, there are few methods to observe Mg^2+^ in mitochondria in intact cells. Here, we have developed a novel Mg^2+^–selective fluorescent probe, KMG-301, that is functional in mitochondria. This probe changes its fluorescence properties solely depending on the Mg^2+^ concentration in mitochondria under physiologically normal conditions. Simultaneous measurements using this probe together with a probe for cytosolic Mg^2+^, KMG-104, enabled us to compare the dynamics of Mg^2+^ in the cytosol and in mitochondria. With this method, carbonyl cyanide *p*-(trifluoromethoxy) phenylhydrazone (FCCP)–induced Mg^2+^ mobilization from mitochondria to the cytosol was visualized. Although a FCCP–induced decrease in the Mg^2+^ concentration in mitochondria and an increase in the cytosol were observed both in differentiated PC12 cells and in hippocampal neurons, the time-courses of concentration changes varied with cell type. Moreover, the relationship between mitochondrial Mg^2+^ and Parkinson's disease was analyzed in a cellular model of Parkinson's disease by using the 1-methyl-4-phenylpyridinium ion (MPP^+^). A gradual decrease in the Mg^2+^ concentration in mitochondria was observed in response to MPP^+^ in differentiated PC12 cells. These results indicate that KMG-301 is useful for investigating Mg^2+^ dynamics in mitochondria. All animal procedures to obtain neurons from Wistar rats were approved by the ethical committee of Keio University (permit number is 09106-(1)).

## Introduction

The magnesium ion (Mg^2+^) is an essential cation for maintaining proper cellular activities. For example, Mg^2+^ is an essential cofactor for hundreds of enzymes [1], [2], the intracellular concentration of Mg^2+^ ([Mg^2+^]_i_) regulates several types of ion channels [3], [4], and Mg^2+^ plays an important role in energy metabolism [5], [6]. [Mg^2+^]_i_ is usually maintained in the range of 0.5–0.7 mM, not only by Mg^2+^ transport across the cell membrane, but also by intracellular buffering and compartmentation into organelles in the majority of mammalian cells [7]. In diseased states, or under the administration of certain hormonal stimuli or cAMP signaling agents, remarkable alterations in [Mg^2+^]_i_ are observed [2], [8], [9]. Therefore, it is important to investigate cellular Mg^2+^ transport and concentration changes in the cytosol and in organelles to better understand intracellular signaling and the pathogenesis of some types of diseases. Because of the absence of Mg^2+^ selective indicators, however, the changes in [Mg^2+^]_i_ in cells and organelles are still not well understood. Commercially available fluorescent Mg^2+^ probes are also sensitive to changes in the intracellular Ca^2+^ concentration, and therefore, those probes are often used as Ca^2+^ indicators [10], [11], [12]. In our group, novel Mg^2+^ selective fluorescent probes, the KMG series, have been developed [13], [14]. These probes are selective for Mg^2+^ and other ions present at physiological concentrations have no effect on their fluorescence. Using these probes, changes in [Mg^2+^]_i_ were measured in many types of cells [15], [16], [17], and it was demonstrated that mitochondria play an important role in intracellular Mg^2+^ dynamics [18], [19].

Mitochondria are the organelles responsible for the cellular energy metabolism, and it was reported that ATP synthesis in mitochondria is regulated by the Mg^2+^ concentration in mitochondria [20]. Mitochondria accumulate Mg^2+^ using their inner membrane potential via Mrs2, which is a Mg^2+^–selective channel expressed in the mitochondrial inner membrane [21], [22]. Mitochondria also transport Mg^2+^ bound to ATP via the ATP–Mg/P_i_ carrier in the mitochondrial inner membrane. This exchanger mediates the electroneutral influx and efflux of ATP–Mg in exchange for P_i_ [23]. Because the depolarization of the mitochondrial inner membrane potential induces the release of Mg^2+^ from mitochondria [18], it is likely that the dysfunction of mitochondria and the disorder of cellular Mg^2+^ regulation are strongly related. Actually, such malfunctions are reported in many neuronal diseases, such as migraine, Alzheimer's disease and Parkinson's disease (PD) [24], [25], [26], [27]. These evidences indicate that the investigation of Mg^2+^ concentration changes in mitochondria is important to understand the detailed mechanisms in intracellular signaling and pathogenesis of such diseases. However, few techniques are available for investigating magnesium concentration changes in mitochondria.

In this study, we have developed a novel Mg^2+^–selective fluorescent probe based on the rhodamine skeleton (KMG-301), and shown that it is functional inside mitochondria in intact cells. Using this probe, Mg^2+^ transport across the mitochondrial membrane and Mg^2+^ concentration changes in the early phases of a cellular model of PD were analyzed.

## Results and Discussion

### Molecular design

**Figure 1:**
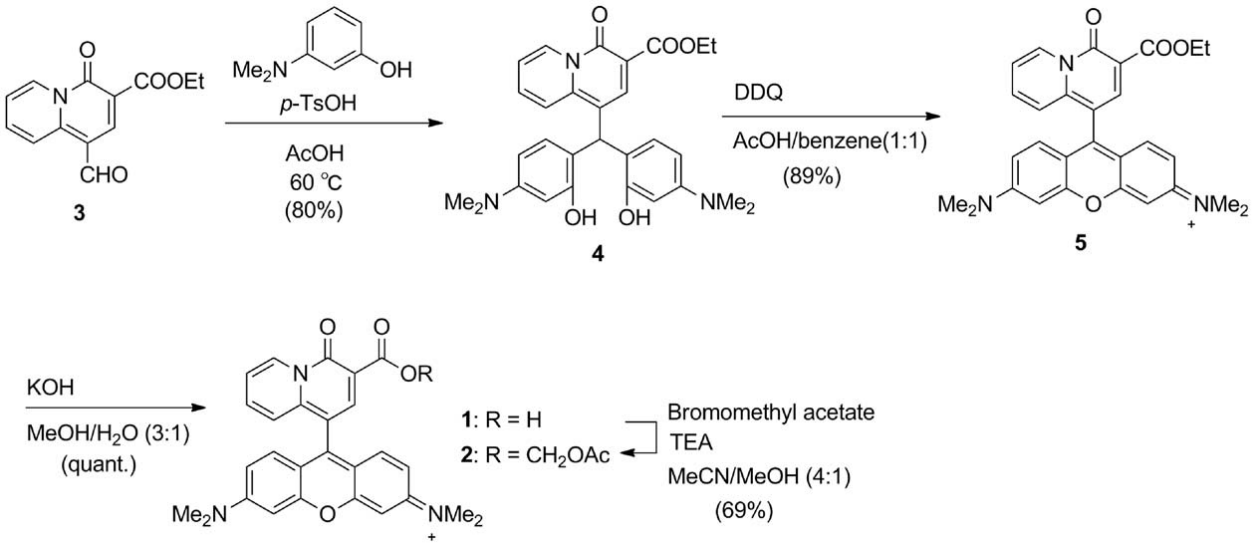
Organic synthesis of KMG-301.

In this work, a Mg^2+^–selective fluorescent probe that is functional in mitochondria was designed and synthesized. The rhodamine skeleton was selected as the chromophore, and a charged-beta-diketone moiety as the binding site for Mg^2+^ coordination. Rhodamine with its cationic charge is known to localize to mitochondria because of their negative membrane potential [28]. Our previous work has shown that the charged-beta-diketone binding site has high selectivity for Mg^2+^ [29]. Several novel Mg^2+^–selective fluorescent probes with the charged-bata-diketone binding site introduced in the form of an α-keto acid have been developed [13], [14], [30]. These probes enable the measurement of [Mg^2+^]_i_ with high selectivity and sensitivity, whereas commercially available Mg^2+^ indicators are less selective over Ca^2+^ [31]. Since Ca^2+^ concentrations in mitochondria are higher than in the cytosol and can vary over a wide range [32], it is important that Mg^2+^ indicators used in mitochondria are not affected by these Ca^2+^ concentration changes. Therefore, the highly Mg^2+^–selective charged-beta-diketone binding site was conjugated to the 9 position of the rhodamine skeleton, resulting in the novel probe KMG-301. This structure shows photoinduced electron transfer (PET)–type OFF-ON responses in the presence of Mg^2+^ ions. The synthetic scheme is shown in Figure 1.

### Molecular properties of KMG-301

**Figure 2:**
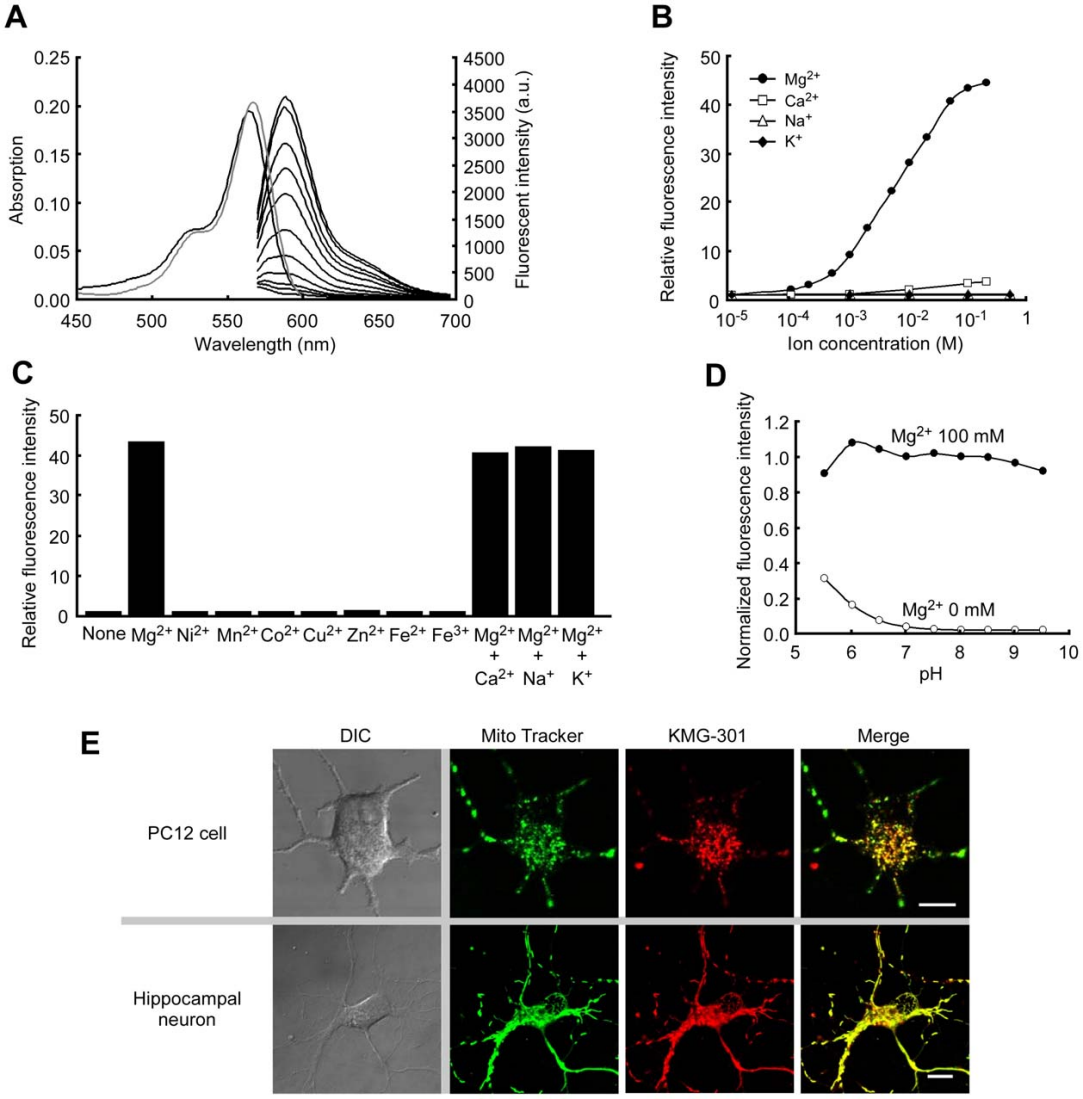
Properties of KMG-301. (A) Absorbance and normalized fluorescence emission spectra of KMG-301 at different Mg^2+^ concentrations. Spectra were measured at a probe concentration of 5 µM at pH 7.2 (100 mM HEPES buffer). Mg^2+^ concentrations are 0 (black line) and 100 mM (gray line) for absorbance spectra, and from 0 mM to 100 mM for fluorescence spectra (excitation 540 nm). (B) Relative fluorescence intensities of KMG-301 in the presence of different cations in concentrations ranging from 0.1 to 200 mM (Mg^2+^, Ca^2+^), and from 0.1 to 500 mM (Na^+^, K^+^) normalized by the value in ion–free solution. (C) Metal ion selectivity of KMG-301 at physiological concentrations. Ni^2+^, Mn^2+^, Co^2+^, Cu^2+^, Zn^2+^, Fe^2+^ and Fe^3+^ were added at 1 µM. Ca^2+^ was added at 1 mM. Na^+^, K^+^ and Mg^2+^ were added at 100 mM. The concentrations were chosen according to the physiological conditions. Fluorescence intensities were normalized by the value in ion–free solution. (D) Effect of the pH on the fluorescence intensity of KMG-301 was measured at pH 5.5–6.5 (in 100 mM MES buffer) and pH 7.0–9.5 (in 100 mM HEPES buffer), with 0 mM or 100 mM of Mg^2+^. The fluorescence intensities were normalized by the value at pH 7.0 with 100 mM of Mg^2+^. (E) Localization of KMG-301 to mitochondria was confirmed in differentiated PC12 cells (upper) and rat hippocampal neurons (bottom). The localization of the fluorescence of KMG-301 (red) and MitoTracker Green FM (green) were corresponded to each other in both cells (merge). The scale bars indicate 10 µm.

The absorbance and fluorescence spectra of KMG-301 were measured at a probe concentration of 5 µM in HEPES buffer (pH 7.2). Whereas 100 mM of Mg^2+^ did not alter the absorbance spectrum of KMG-301, it induced a 45-fold increase in fluorescence emission intensity compared to the ion-free condition (Figure 2A). The dissociation constant of this probe for Mg^2+^ was found to be 4.5 mM, which is a suitable value for detecting Mg^2+^ in mitochondria. The fluorescence quantum yield (Φ_fl_) and the molar extinction coefficient (ε) were determined as 0.15 and 42100 M^−1^cm^−1^, respectively. Although KMG-301 showed a 3-fold fluorescence increase in the presence of 100 mM Ca^2+^, no fluorescence increase was observed at 1 mM Ca^2+^ (Figure 2B). Therefore, the physiologically relevant Ca^2+^ concentrations (∼100 µM) do not affect the fluorescence of KMG-301. Physiological concentration ranges of Na^+^ and K^+^ also have no effect on the fluorescence of KMG-301, both in the absence or presence of Mg^2+^ (Figure 2B and C). Moreover, other divalent cations did not influence the fluorescence signal at physiological concentrations (Figure 2C). KMG-301 was found to be sensitive to 1 mM of Ni^2+^ and Zn^2+^ (Figure S1). But in a physiological environment, concentrations of divalent cations other than Mg^2+^ and Ca^2+^ are below 1 µM whereas that of Mg^2+^ is 0.5–0.7 mM. Therefore, it can be concluded that KMG-301 is only sensitive to Mg^2+^ under intracellular and intramitochondrial conditions. The ion-free and Mg-bound fluorescence emission peaks of KMG-301 showed only a weak response to pH in the range of pH 6.5–9 (Figure 2D). The mitochondrial pH value is considered to be around 8.6. As a consequence, it can be concluded that pH (>6.0) and any other cations do not interfere with the measurement of Mg^2+^ concentrations using KMG-301 under physiologically normal intracellular conditions.

The localization of KMG-301 to mitochondria was confirmed in differentiated PC12 cells and hippocampal neurons. KMG-301 in the acetoxy methyl esterified form (KMG-301AM) (Figure 1) was applied to these cells. The localization of KMG-301 corresponded with that of MitoTracker Green FM (Figure 2E). This data indicates that KMG-301 was successfully localized in mitochondria in intact cells.

### KMG-301 functionality in mitochondria

**Figure 3:**
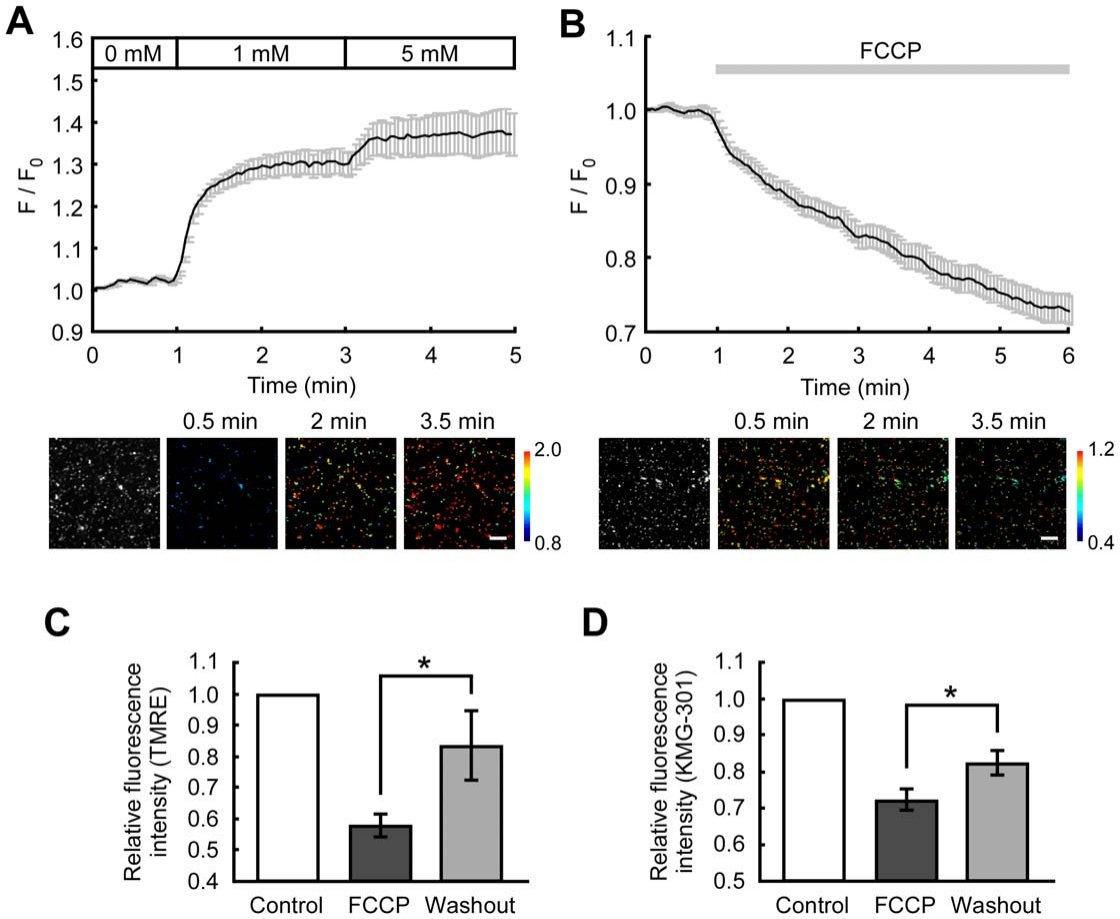
KMG-301 detects the changes in Mg^2+^ concentration in isolated mitochondria. (A) Time-course and pseudo-colored images of the change in the fluorescence of KMG-301 in isolated mitochondria stained with KMG-301AM. Stepwise increases in the extramitochondrial Mg^2+^ concentration induced increases in the fluorescence of KMG-301 in mitochondria. This indicates that Mg^2+^ absorption in mitochondria was measured successfully with KMG-301. The scale bar indicates 10 µm. (B) Time-course and pseudo-colored images of the change in the fluorescence of KMG-301 in response to 5 µM FCCP. The FCCP–induced release of Mg^2+^ from mitochondria was observed successfully. (C) Mean fluorescence intensities of TMRE before stimuli, 5 min after FCCP treatment and 10 min after washout in isolated mitochondria from PC12 cells. The membrane potential partially recovered after washout of FCCP. (D) Mean fluorescence intensities of KMG-301 before stimuli, 5 min after FCCP treatment and 10 min after washout. Fluorescence of KMG-301 was increased upon repolarization of the mitochondrial membrane potential. This data indicates that the fluorescence of KMG-301 is responding to the changes in [Mg^2+^]_mito_. The error bars indicate SEM.

To confirm that KMG-301 is able to indicate changes in mitochondrial Mg^2+^ concentration ([Mg^2+^]_mito_), isolated mitochondria from PC12 cells were stained with KMG-301AM. Increasing the Mg^2+^ concentration around mitochondria from 0 mM to 1 mM induced a noticeable increase of KMG-301 fluorescence. A further increase in Mg^2+^ concentration around mitochondria from 1 mM to 5 mM induced a further increase in KMG-301 fluorescence (Figure 3A). In previous studies, we have demonstrated that carbonyl cyanide *p*-(trifluoromethoxy) phenylhydrazone (FCCP) induces Mg^2+^ release from mitochondria in PC12 cells [18]. Administration of FCCP induced a gradual decrease in KMG-301 fluorescence in mitochondria isolated from PC12 cells (Figure 3B). The time-course of this KMG-301 fluorescence decrease was similar to that of the FCCP-induced decrease in mitochondrial membrane potential in mitochondria isolated from PC12 cells (Figure S2). In mitochondria isolated from rat brain, FCCP also induced a gradual decrease in the fluorescence of KMG-301, whereas it induced a rapid decrease in the fluorescence of TMRE (Figure S2). It was confirmed that the FCCP-induced decrease in KMG-301 fluorescence was not caused by leaking of the dye from mitochondria. When the membrane potential partially recovered after washout of FCCP from mitochondria isolated from PC12 cells, the KMG-301 fluorescence also recovered significantly (Figure 3C and D). Mitochondria accumulated Mg^2+^ depending on the increase in their membrane potential, and KMG-301 could successfully detect the increase in Mg^2+^ concentration in mitochondria. This data allows to conclude that KMG-301 changes its fluorescence in response to the changes in [Mg^2+^]_mito_.

### KMG-301 responds to Mg^2+^ concentration changes in mitochondria in intact cells

**Figure 4:**
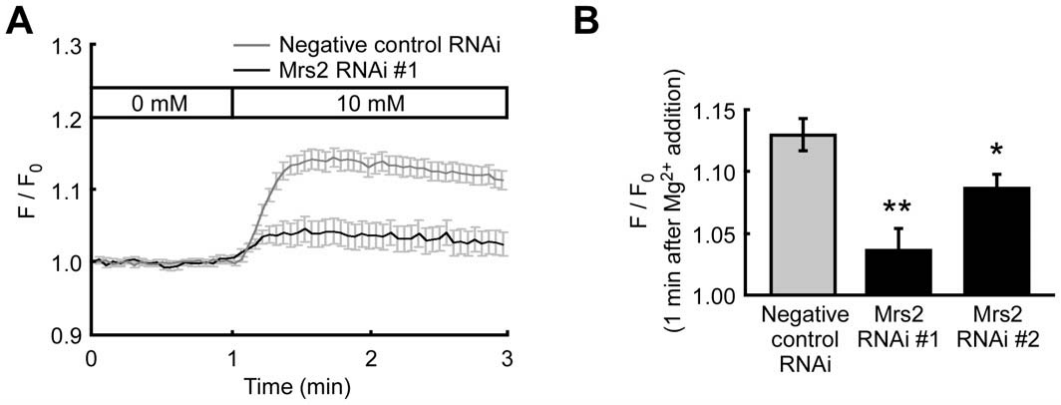
KMG-301 is functional in mitochondria in living cells. (A) Time-course of the changes in the fluorescence of KMG-301 in PC12 cells expressing negative control miR RNAi (gray line) and miR RNAi for Mrs2 (black line). An increase in the extracellular Mg^2+^ concentration from 0 mM to 10 mM induced an increase in the fluorescence of KMG-301 in PC12 cells expressing negative control miR RNAi. This increase in fluorescence was significantly suppressed in PC12 cells expressing miR RNAi for Mrs2. (B) Comparison of the relative fluorescence intensities of KMG-301 1 minute after the increase in extracellular Mg^2+^ concentration. Both two miR RNAis significantly inhibited the increase in fluorescence of KMG-301. This data indicates that KMG-301 was internalized in mitochondria and responded to changes in [Mg^2+^]_mito_. The error bars indicate SEM. * indicates *p*<0.05, and ** indicates *p*<0.01.

Mrs2 is known as a Mg^2+^–selective channel expressed in the mitochondrial inner membrane, and mitochondria accumulate Mg^2+^ via this channel depending on an electrochemical gradient [33]. To confirm that KMG-301 accumulates inside mitochondria in intact cells, changes in probe fluorescence in normal cells and in cells with suppressed Mrs2 expression levels were compared. Suppression of the Mrs2 expression levels was performed by transfection of a vector encoding both emerald GFP (EmGFP) and a miR RNAi for Mrs2 to PC12 cells (Figure S3). Because miR RNAi–expressing cells also express EmGFP, fluorescence changes of KMG-301 were measured only in EmGFP expressing cells. An increase in the extracellular Mg^2+^ concentration from 0 mM to 10 mM induced a remarkable increase in cytosolic Mg^2+^ concentration ([Mg^2+^]_cyto_) and a fluorescence increase of KMG-301 (Figures S4 and 4A gray line). This change in fluorescence of KMG-301 was caused by the change in [Mg^2+^]_mito_ not by that in [Mg^2+^]_cyto_ because no significant increase in fluorescence was observed in mitochondria-free areas (Figure S5). This increase in fluorescence of KMG-301 was significantly suppressed in miR RNAi–expressing cells (Figure 4A black line and B). This data not only indicates that KMG-301 was successfully internalized in mitochondria and detected the changes in [Mg^2+^]_mito_ in intact cells, but also that Mrs2 plays an important role in Mg^2+^ accumulation in mitochondria.

**Figure 5:**
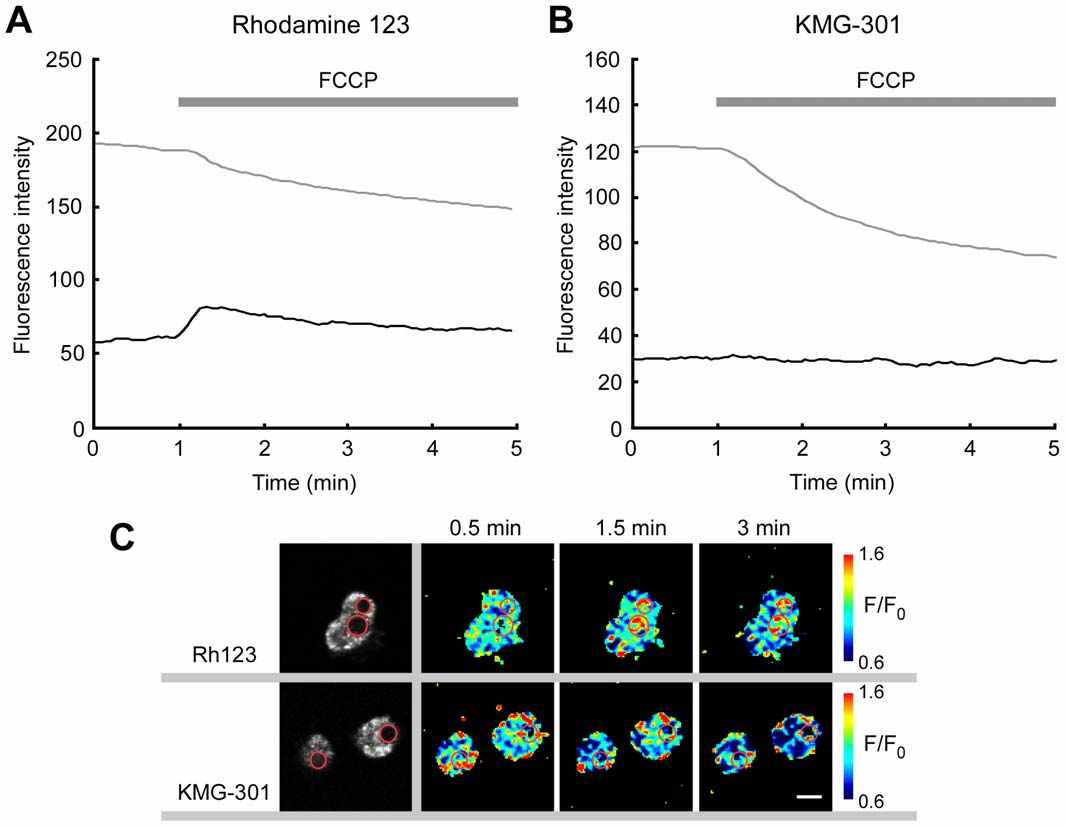
KMG-301 was not released from mitochondria upon depolarization of their membrane potential. (A) FCCP induced a decrease in fluorescence of rhodamine 123 in the cell body (presence of mitochondria, gray line). On the other hand, in the nucleus area (no mitochondria area), an increase in fluorescence intensity was observed (black line). It is caused by the release of the probe from mitochondria to the cytosol. These lines show the time-course average of 60 cells. (B) Whereas FCCP also induced a decrease in fluorescence of KMG-301 in the cell body (gray line), no increase in fluorescence was observed in the nucleus area (no mitochondria area, black line). This data indicates that KMG-301 is not released from mitochondria depending on the depolarization of their membrane potential. These lines show the time-course average of 43 cells. (C) Fluorescence images and pseudo-colored images (F/F_0_) of non-differentiated PC12 cells stained with Rh123 and KMG-301. Upon FCCP administration, increases in fluorescence in the nucleus area were observed only in Rh123 loaded cells. Red circles indicate the nucleus areas. The scale bar indicates 10 µm.

It was also confirmed that KMG-301 is not released from mitochondria upon depolarization of the mitochondrial membrane potential. Rhodamine123 was used in a control experiment. Rhodamine 123 is a cell–permeable cationic fluorescent dye and widely used as a marker for mitochondria. This probe accumulates in the matrix of mitochondria dependent on their membrane potential. Upon depolarization of the mitochondrial membrane potential, Rhodamine 123 is released into the cytosol and the extracellular medium. Whereas FCCP induced a decrease in the fluorescence intensity of Rhodamine 123 in the cell body (presence of mitochondria), it induced an increase in the nucleus area (no mitochondria; Figure 5A and C). This indicates that the probe released from mitochondria caused an increase in the probe concentration in the cytosol and the nucleus, accompanied by a fluorescence increase in the same areas. On the other hand, whereas FCCP also induced a decrease in the fluorescence intensity of KMG-301 in the cell body, no increase in fluorescence was observed in the nucleus area of PC12 cells (Figure 5B and C). This indicates that KMG-301 accumulated in mitochondria and was not released from mitochondria by depolarization of the mitochondrial membrane potential. KMG-301AM successfully accumulates in mitochondria and is then hydrolyzed to KMG-301. Since the mitochondrial membrane is impermeable to KMG-301, it is not released upon depolarization of the mitochondrial membrane potential. Therefore, it can be concluded that KMG-301 is a Mg^2+^–selective fluorescent probe functional in mitochondria in intact cells. To the best of our knowledge, this is the first reported method for visualization of changes in [Mg^2+^]_mito_ in intact cells.

### Imaging of intracellular Mg^2+^ mobilization from mitochondria

Because the excitation and emission spectra of KMG-301 are different from those of the cytosolic Mg^2+^ probe KMG-104, it is possible to simultaneously observe the fluorescence of both probes. The interaction between the cytosolic and mitochondrial Ca^2+^ concentrations was observed and analyzed with simultaneous use of fura-2 and rhod-2 [34], [35]. These studies have clarified the contribution of mitochondria to Ca^2+^–buffering and Ca^2+^ mobilization. In this study, Mg^2+^ transport across the mitochondrial membrane was visualized by simultaneous application of KMG-104AM and KMG-301AM.

**Figure 6:**
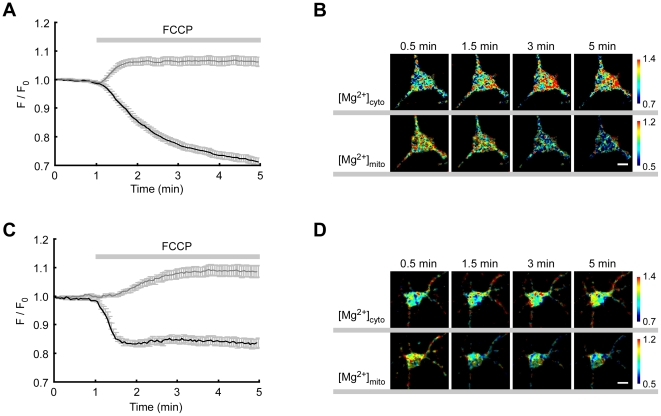
Simultaneous measurements of cytosolic and mitochondrial Mg^2+^ concentration changes. (A) Time-course of the changes in [Mg^2+^]_cyto_ (gray line) and [Mg^2+^]_mito_ (back line) was measured with KMG-104AM and KMG-301AM, respectively, in differentiated PC12 cells. Administration of 5 µM FCCP induced a rapid increase in [Mg^2+^]_cyto_ and a gradual decrease in [Mg^2+^]_mito_. (B) Pseudo–colored images of changes in [Mg^2+^]_cyto_ (upper) and [Mg^2+^]_mito_ (bottom) were obtained at the indicated times. (C) Time-course of the changes in [Mg^2+^]_cyto_ (gray line) and [Mg^2+^]_mito_ (back line) in hippocampal neurons. Administration of 5 µM FCCP induced a gradual increase in [Mg^2+^]_cyto_ and a rapid decrease in [Mg^2+^]_mito_. (D) Pseudo–colored images of changes in [Mg^2+^]_cyto_ (upper) and [Mg^2+^]_mito_ (bottom) were obtained at the indicated times. The error bars indicate SEM. The scale bars indicate 10 µm.

In our previous study, FCCP–induced Mg^2+^ release from mitochondria was shown by comparing the changes in [Mg^2+^]_cyto_ around mitochondria with those in areas with no mitochondria [18]. Because KMG-301 allows to measure the changes in [Mg^2+^]_mito_ directly, in this study, the changes in [Mg^2+^]_cyto_ and [Mg^2+^]_mito_ were measured Moreover, Mg^2+^ dynamics were compared in differentiated PC12 cells and in hippocampal neurons. In differentiated PC12 cells, [Mg^2+^]_cyto_ increased for 1 min after FCCP administration and maintained a high value, whereas [Mg^2+^]_mito_ decreased for more than 4 minutes after FCCP treatment (Figures 6A and B). In hippocampal neurons, [Mg^2+^]_mito_ decreased only for 30 seconds after FCCP treatment and kept its low value, although [Mg^2+^]_cyto_ increased for about 3 minutes (Figures 6C and D). Although the FCCP–induced decrease in [Mg^2+^]_mito_ and increase in [Mg^2+^]_cyto_ were observed both in differentiated PC12 cells and in hippocampal neurons, the time-course of concentration changes varied with the cell type. Because the time-courses of the depolarization of the mitochondrial membrane potential also varied with these cell types (Figure S6), it could be one of the reasons for the variation in Mg^2+^ transport. Moreover, these simultaneous measurements of changes in [Mg^2+^]_cyto_ and [Mg^2+^]_mito_ revealed that the time-course of the [Mg^2+^]_cyto_ increase and the [Mg^2+^]_mito_ decrease is not symmetric. Since the Mg^2+^ concentration is regulated by Mg^2+^ entry, Mg^2+^ efflux, buffering and compartmentation [7], [36], it seems that those differences in the Mg^2+^ concentration changes are caused by the cell type dependent differences in the balance of these mechanisms and the intracellular compartments.

Mg^2+^ entry into cells is mediated primarily by ion channels. Recently, many types of Mg^2+^–permeable channels have been identified [4], [37]. Some of them alter their expression levels or localization based on the environmental Mg^2+^ concentration. Buffering and muffling of increased [Mg^2+^]_cyto_ are carried out mainly by Mg^2+^ extrusion via the Na^+^/Mg^2+^ exchanger and Mg^2+^ binding to ATP [38]. In PC12 cells, Mg^2+^ is extruded via the Na^+^/Mg^2+^ exchanger after FCCP administration [17]. It was estimated that almost 1/3 of Mg^2+^ muffling is due to Mg^2+^ binding to ATP and another 1/3 is due to Na^+^/Mg^2+^ exchange in leech neurons [39]. One part of the remaining 1/3 is expected to result from sequestration by mitochondria. Here, it was demonstrated that mitochondria also accumulate Mg^2+^ (Figure 4). Mrs2 plays an important role in this accumulation, because knockdown of this protein significantly suppressed the increase in [Mg^2+^]_mito_ (Figure 4). This result is consistent with a study on isolated mitochondria [40]. It was also suggested that the endoplasmic reticulum accumulates Mg^2+^ [41]. The golgi apparatus and endosomes have Mg^2+^ transport proteins [37]. It could be possible that these organelles also participate in the Mg^2+^ buffering mechanism. [Mg^2+^]_mito_ is regulated by Mrs2 and the ATP-Mg/P_i_ carrier in the mitochondrial inner membrane. This antiporter electroneutrally mediates Mg^2+^ transport depending on the concentration gradients of P_i_ and ATP [23]. The ATP concentration also affects the Mg^2+^ concentration [38]. Moreover, it was reported that unidentified Mg^2+^–binding proteins exist in the intermembrane space of mitochondria [42]. A balance of all those mechanisms controls the Mg^2+^ concentrations in the cytosol and in mitochondria with the possibility of further mechanisms being involved. Simultaneous measurements of [Mg^2+^]_cyto_ and [Mg^2+^]_mito_ enable the comparison of the Mg^2+^ dynamics in these two compartments and revealed that a collapse of the mitochondrial membrane potential induces the perturbation of Mg^2+^ concentrations in the cytosol and mitochondria.

### PD inducible substance causes Mg^2+^ decrease in mitochondria

**Figure 7:**
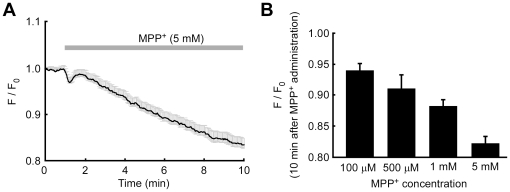
Putative PD inducible substance caused Mg^2+^ release from mitochondria. (A) Time-course of the change in [Mg^2+^]_mito_ was measured with KMG-301 in differentiated PC12 cells. Administration of 5 mM MPP^+^ induced a gradual decrease of [Mg^2+^]_mito_. The error bars indicate SEM. (B) Comparison of the relative fluorescence intensities of KMG-301 10 minutes after the administration of MPP^+^ at the indicated concentrations. The [Mg^2+^]_mito_ decreased in a dose-dependent manner. The error bars indicate SEM.

In dopaminergic cells, 1-methyl-4-phenylpyridinium ion (MPP^+^)–induced cell death is an excellent model of PD. Because PC12 cells are dopaminergic cells, MPP^+^–induced cell death in PC12 cells has been widely used for investigations of the mechanisms of neurodegeneration in PD [43], [44]. Since a mitochondria dysfunction was reported in PD patients and in MPP^+^–treated PC12 cells [44], [45], it is likely that [Mg^2+^]_mito_ in PD patients and MPP^+^–treated cells were perturbed. To confirm this hypothesis, changes in [Mg^2+^]_mito_ in response to MPP^+^ were measured in differentiated PC12 cells. After administration of MPP^+^, [Mg^2+^]_mito_ decreased gradually (Figure 7A). The time-course of Mg^2+^ release from mitochondria was similar to that of the depolarization in the mitochondrial membrane potential (Figure S7). MPP^+^ in the range of 0.1–5 mM caused a Mg^2+^ release from mitochondria in a dose–dependent manner (Figure 7B). KMG-301 was bleached less than 5% in 10 minutes. In differentiated PC12 cells, 0.5–10 mM MPP^+^ induces cell death in a dose–dependent manner in 24 hours [46]. MPP^+^–induced Mg^2+^ release from mitochondria was observed in a concentration range consistent with the previous report. Because MPP^+^ acts as an inhibitor of the mitochondrial electron transfer chain, the mitochondrial membrane potential collapsed in MPP^+^–treated PC12 cells [47]. Therefore, it is likely that MPP^+^–induced Mg^2+^ release from mitochondria was caused by a decrease in the mitochondrial membrane potential, which is the same mechanism as in the case of FCCP–induced Mg^2+^ release from mitochondria. Because a decline of mitochondrial function is also observed in PD patients [45], this Mg^2+^ release from mitochondria might also occur in the neurons of PD patients. As described above, Mg^2+^ efflux from mitochondria results in Mg^2+^ extrusion from cells via the Na^+^/Mg^2+^ exchanger, and thus in a decrease of cellular Mg^2+^. These observations imply that the cellular Mg^2+^ content is decreased in the early phase of MPP^+^–induced cell death in PC12 cells and during neurodegeneration in PD.

In some studies, the deficiency of Mg^2+^ and PD were found to be closely related. PD–like dopaminergic neuronal loss was observed in the rat brain after exposure to low magnesium intake over generations [48]. It was also reported that the Mg^2+^ concentration is lowered in the brain of PD patients [26]. These reports suggest that low Mg^2+^ uptake to neurons and/or Mg^2+^ efflux from neurons are related to PD. Because Mg^2+^ exerts a preventive effect against MPP^+^–induced neuronal degeneration in rat dopaminergic neurons [49], the loss of cellular Mg^2+^ seems to accelerate the process of MPP^+^–induced cell death and neuronal degeneration in PD. Thus, Mg^2+^ release from mitochondria reported here seems to be a crucial event in the model of PD.

## Materials and Methods

### Ethics statement

All animal procedures were approved by the ethical committee of Keio University (permit number is 09106-(1)).

### Synthesis

The organic synthesis of KMG-301 is described in the supporting information (Text S1).

### Measurement of probe properties

Absorption measurements were performed on a U-2001 spectrophotometer (HITACHI, Tokyo, Japan). For the determination of the fluorescence quantum yield (Φ_fl_), rhodamine101 (Φ_fl_ = 0.96) was used as fluorescence standard. Φ_fl_ and ε were measured in 100 mM HEPES buffer (pH 7.2) containing 100 mM Mg^2+^. Ion selectivity, pH sensitivity and fluorescence spectra were measured using a SPECTRAMAX GEMINI XS fluorescence microplate reader (Molecular Devices, Mountain View, CA, USA). The fluorescence of 5 µM KMG-301 was measured in 100 mM HEPES buffer (pH 7.2) with or without metal ions to estimate the ion selectivity. The effect of pH on the fluorescence of 5 µM KMG-301 was measured in 100 mM MES buffer (pH 5.5–7.0) or 100 mM HEPES buffer (pH 7.5–9.5) with or without 100 mM Mg^2+^. Excitation was performed at 540 nm and fluorescence emission was measured from 570 to 700 nm. The fluorescence emission intensity at 590 nm was used as the signal in the various experiments.

### Cell culture

The primary culture of hippocampal neurons was prepared from day 17–19 embryonic Wistar rats (Charles River Laboratories Japan, Tokyo, Japan). Hippocampi were excised and submerged in ice-cold PBS(-). The hippocampal neurons were dissociated using dissociation solution (Sumitomo Bakelite Co., Tokyo, Japan), and plated on poly-D-lysine (PDL; Sigma-Aldrich, St. Louis, MO, USA)–coated glass bottom dishes (Iwaki, Tokyo, Japan), and cultured in neurobasal medium supplemented with B-27, 2 mM L-glutamine, 50 U/ml penicillin and 50 µg/ml streptomycin (all from Invitrogen, Carlsbad, CA, USA). Cultures were maintained at 37°C in a humidified atmosphere of 5% CO_2_. Cells were cultured for a minimum of seven days before experimental use.

PC12 cells were obtained from RIKEN Tsukuba Institute, and cultured in DMEM containing 10% horse serum, 5% FBS, 50 U/ml penicillin and 50 µg/ml streptomycin. For experimental use, cells were cultured on PDL–coated glass bottom dishes. In some experiments, cells were differentiated by culturing in serum–free medium with 50 ng/ml NGF for 5 days.

### Isolation of mitochondria

Mitochondria were isolated from PC12 cells and rat brain (E19) using the Mitochondria Isolation Kit (BioChain Institute Inc., Gibbstown, NJ, USA). In short, cells were suspended in Mitochondria Isolation Buffer and homogenized with a Dounce homogenizer on ice. The homogenate was centrifuged 600×g for 10 min at 4°C, and the supernatant was further centrifuged at 12,000×g for 15 min at 4°C. The pellet was suspended in Mitochondria Storage Buffer and placed on Cell-Tak (BD Biosciences, Franklin Lakes, NJ, USA)–coated glass bottom dishes, and incubated 30–90 min on ice to embed on glass.

### Fluorescence measurements

For optical imaging, 20 µM KMG-301AM was applied to cells in Hanks' balanced salt solutions (HBSS) containing (in mM): NaCl, 137; KCl, 5.4; CaCl_2_, 1.3; MgCl_2_, 0.5; MgSO_4_, 0.4; Na_2_HPO_4_, 0.3; KH_2_PO_4_, 0.4; NaHCO_3_, 4.2; D-glucose, 5.6; HEPES, 5 (pH adjusted to 7.4 with NaOH) for 10 min on ice, so that hydrolysis of the acetoxymethyl ester by esterase present in the cytosol would be avoided [50]. Then, the cells were washed twice with HBSS and incubated for 15 min at 37°C to allow for complete hydrolysis of the acetoxymethyl ester form in mitochondria. For the simultaneous use of KMG-104AM and KMG-301AM, cells were incubated with 5 µM KMG-104AM in HBSS for 30 min at 37°C, and then stained with KMG-301AM. For simultaneous use of KMG-301AM and MitoTracker Green FM, KMG-301–loaded cells were incubated with 5 nM MitoTracker Green FM for 10 min at 37°C, and then the cells were washed twice with HBSS. Rhodamine 123 was used at 100 nM, which is a non-quenching concentration. Cells were incubated with Rhodamine 123 in HBSS for 30 min at 37°C. Then the cells were washed twice with HBSS and incubated for 15 min in 37°C.

Isolated mitochondria were incubated with 20 µM KMG-301AM in Mitochondria Imaging Buffer (MIB) containing (in mM): KCl, 125; K_2_HPO_4_, 2; MgCl_2_, 1; HEPES, 5; EDTA, 0.02 (pH adjusted to 7.2 with KOH) or Mg^2+^-free MIB (without MgCl_2_) for 20 min at 37°C. Then, mitochondria were washed twice with MIB and further incubated for 15 min at 37°C.

Fluorescence imaging experiments were performed using a confocal laser scanning microscope system (FluoView FV1000; Olympus, Tokyo, Japan) mounted on an inverted microscope (IX81; Olympus) with 40× and 60× oil–immersion objective lenses. KMG-301 was excited at 559 nm from a laser diode, and the signal was observed at 600–700 nm. In the simultaneous measurement, KMG-104 and KMG-301 were excited at 488 nm from an Argon laser and 559 nm, respectively. Fluorescence was separated using a 560 nm dichroic mirror and observed at 500–545 nm and 600–700 nm, respectively. Fluorescent images were acquired and analyzed with the FluoView software package (Olympus). Fluorescence differences were calculated as the mean intensity over a defined region of interest (ROI) containing the cell body of each cell.

### Knockdown of Mrs2 protein

miR RNAi–mediated knockdown was performed using the BLOCK-iT™ PolII miR RNAi Expression Vector Kit with EmGFP (Invitrogen). The miR RNAis sequences are listed in the supporting information. The expression vector was transfected to PC12 cells by electroporation using Neon (Invitrogen). Experiments were performed 4-days after transfection. The expression of miR RNAi was confirmed by the co-expression of EmGFP. In the fluorescence measurements, the fluorescence of KMG-301 was observed only in EmGFP–expressing cells.

### Statistical analyses

Significant differences were determined by using Tukey's test for multiple comparisons. The *p* values less than 0.05 were considered as being significantly different.

## Supporting Information

Figure S1 **Ion selectivity of KMG-301.** The same concentration (1 mM) was used for investigating the relative fluorescence intensity. KMG-301 is also sensitive to Ni^2+^ and Zn^2+^ but not to Ca^2+^, Na^+^ and K^+^ at equimolar concentrations. Undre the physiological condition, the concentrations of Ni^2+^, Mn^2+^, Co^2+^ and Zn^2+^ are less than 1 µM, whereas that of Mg^2+^ is 0.5–0.7 mM. Therefore, KMG-301 is sensitive only to Mg^2+^ under intracellular and intramitchondrial conditions.
(TIF)

Figure S2 **FCCP-induced depolarization and decrease in KMG-301 fluorescence in isolated mitochondria.** (A) Time-course of FCCP-induced depolarization in isolated mitochondria from PC12 cells were measured with TMRE. The mitochondrial membrane potential gradually decreased. (B) Time-course of FCCP-induced depolarization in isolated mitochondria from rat brain. The mitochondrial membrane potential rapidly decreased after administration of FCCP. (C) Time-course of FCCP-induced decrease in KMG-301 fluorescence in isolated mitochondria from the rat brain. The fluorescence of KMG-301 gradually decreased and its time-course is different from the rapid depolarization of the mitochondrial membrane potential. The error bars indicate SEM.
(TIF)

Figure S3 **Knockdown of Mrs2 in PC12 cells.** Relative expression levels of Mrs2 mRNA in PC12 cells transfected with expression vectors for miR RNAi against Mrs2 (Mrs2 RNAi #1 and #2), negative control miR RNAi and non–transfected cells were estimated with real-time PCR. In cells transfected with the miR RNAi against Mrs2, the expression levels of Mrs2 mRNA were suppressed. The efficiency of transfection of the vector to PC12 cells ranged from 40–50%. The efficiencies of suppression varied with the sequences of miR RNAi. Expression levels were measured in 3-4 different samples and compared with that in non-transfected cells. The error bars indicate SEM. * indicates *p*<0.05 estimated by using Tukey's test.
(TIF)

Figure S4 **Mg^2+^ uptake into PC12 cells.** The time-course of the change in [Mg^2+^]_cyto_ (black line) and of the change in fluorescence of KMG-301 (gray line) was simultaneously measured in PC12 cells. Increasing the extracellular [Mg^2+^] from 0 mM to 10 mM induced a remarkable increase in [Mg^2+^]_cyto_ and an increase in the fluorescence of KMG-301([Mg^2+^]_mito_). The error bars indicate SEM.
(TIF)

Figure S5 **The fluorescence intensity of KMG-301 changed in the ROIs of the cell body.** Non-differentiated PC12 cells expressing TagCFP-Mito (purchased from Evrogen, Moscow, Russia) were stained with KMG-301. As shown in Figure 4, an increase in extracellular Mg^2+^ concentration from 0 to 10 mM induced an increase in the fluorescence of KMG-301. Here, the fluorescence intensities of KMG-301 before and after application of 10 mM Mg^2+^ were compared in the ROI of the whole cell body, which contains mitochondria, and in the mitochondria-free area, which shows no fluorescence of TagCFP-Mito. Significant increases in the fluorescence intensity of KMG-301 were observed only in the ROIs of the whole cell body (N = 9, n = 20). The error bars indicate SEM.
(TIF)

Figure S6 **FCCP-induced depolarization of mitochondrial membrane potentials in differentiated PC12 cells and hippocampal neurons.** (A) The time-course of FCCP-induced depolarization in PC12 cells was measured with TMRE. The mitochondrial membrane potential gradually decreased. (B) Time-course of FCCP-induced depolarization in hippocampal neurons. The error bars indicate SEM.
(TIF)

Figure S7 **MPP^+^-induced depolarization of mitochondrial membrane potentials in differentiated PC12 cells.** The time-course of MPP^+^-induced depolarization in differentiated PC12 cells was measured with TMRE. The mitochondrial membrane potential gradually decreased after MPP^+^ application. The error bars indicate SEM.
(TIF)

Text S1 **The organic synthesis of KMG-301 and supporting methods are included in this file.**
(DOC)
