# Supplementary figures and images for: Newly Developed Mg2+–Selective Fluorescent Probe Enables Visualization of Mg2+ Dynamics in Mitochondria
Source: PLoS One. 2011 Aug 16;6(8):e23684. doi: 10.1371/journal.pone.0023684 (PMC3156752; doi:10.1371/journal.pone.0023684)

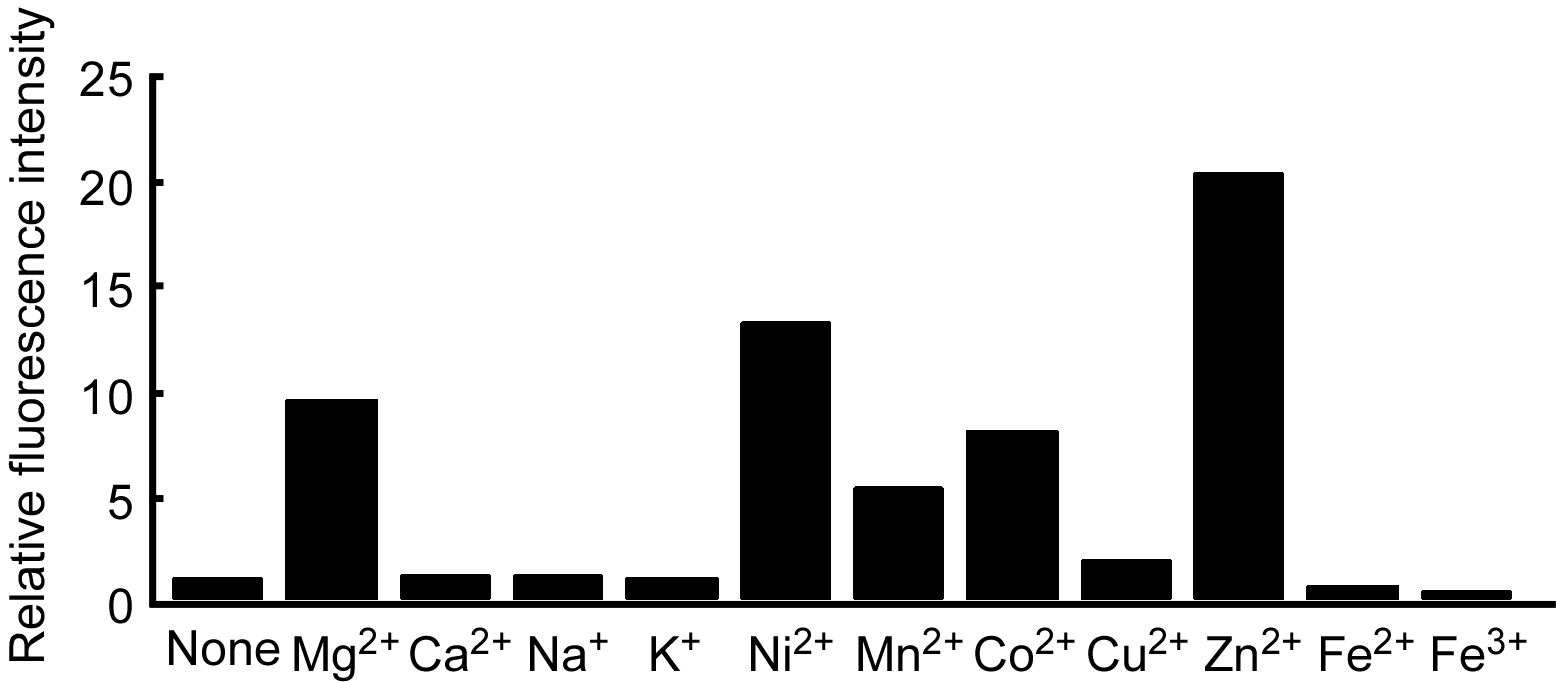

Supplement: Figure S1 — Ion selectivity of KMG-301. The same concentration (1 mM) was used for investigating the relative fluorescence intensity. KMG-301 is also sensitive to Ni2+ and Zn2+ but not to Ca2+, Na+ and K+ at equimolar concentrations. Undre the physiological condition, the concentrations of Ni2+, Mn2+, Co2+ and Zn2+ are less than 1 µM, whereas that of Mg2+ is 0.5–0.7 mM. Therefore, KMG-301 is sensitive only to Mg2+ under intracellular and intramitchondrial conditions. (TIF) [file pone.0023684.s001.tif]

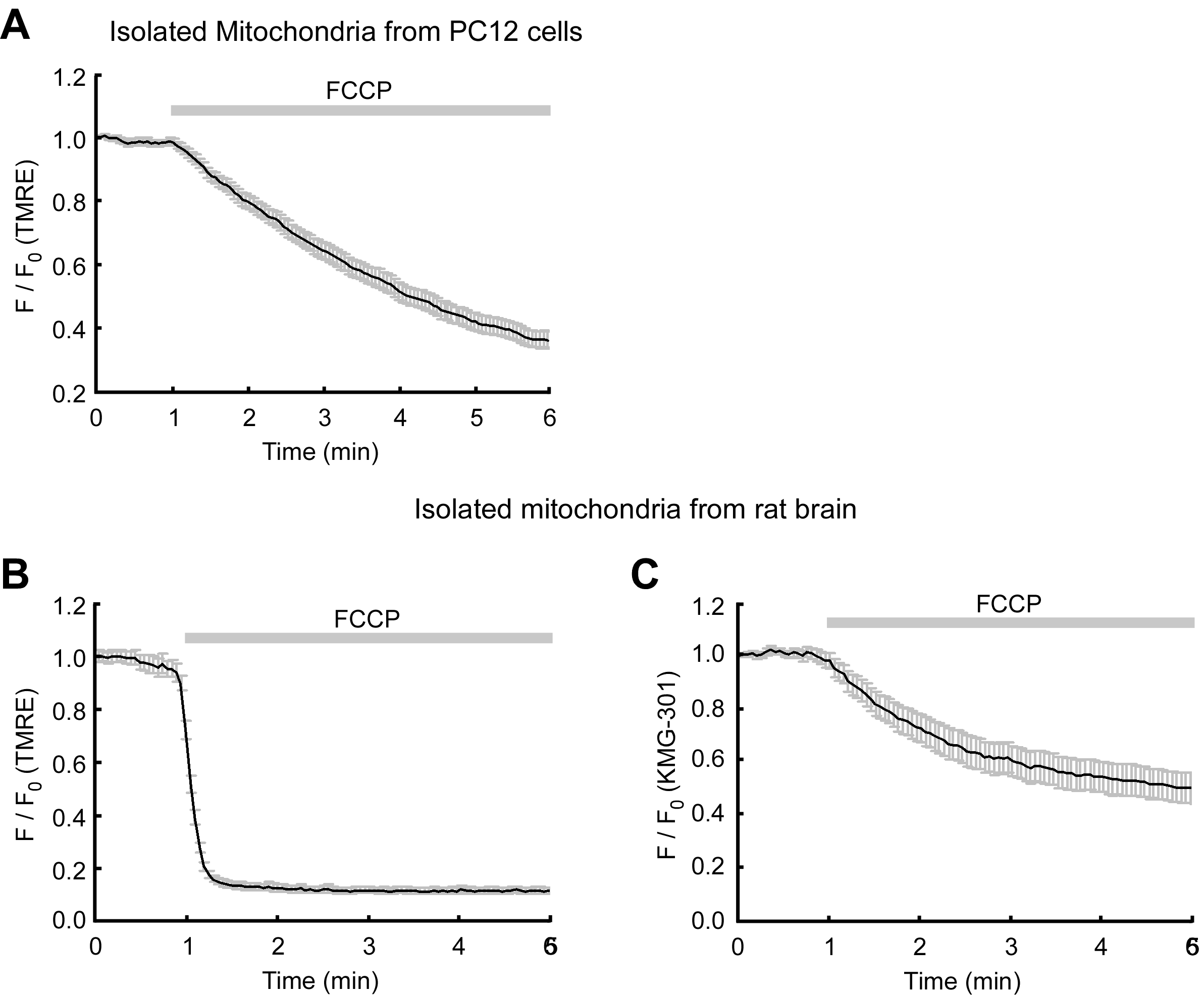

Supplement: Figure S2 — FCCP-induced depolarization and decrease in KMG-301 fluorescence in isolated mitochondria. (A) Time-course of FCCP-induced depolarization in isolated mitochondria from PC12 cells were measured with TMRE. The mitochondrial membrane potential gradually decreased. (B) Time-course of FCCP-induced depolarization in isolated mitochondria from rat brain. The mitochondrial membrane potential rapidly decreased after administration of FCCP. (C) Time-course of FCCP-induced decrease in KMG-301 fluorescence in isolated mitochondria from the rat brain. The fluorescence of KMG-301 gradually decreased and its time-course is different from the rapid depolarization of the mitochondrial membrane potential. The error bars indicate SEM. (TIF) [file pone.0023684.s002.tif]

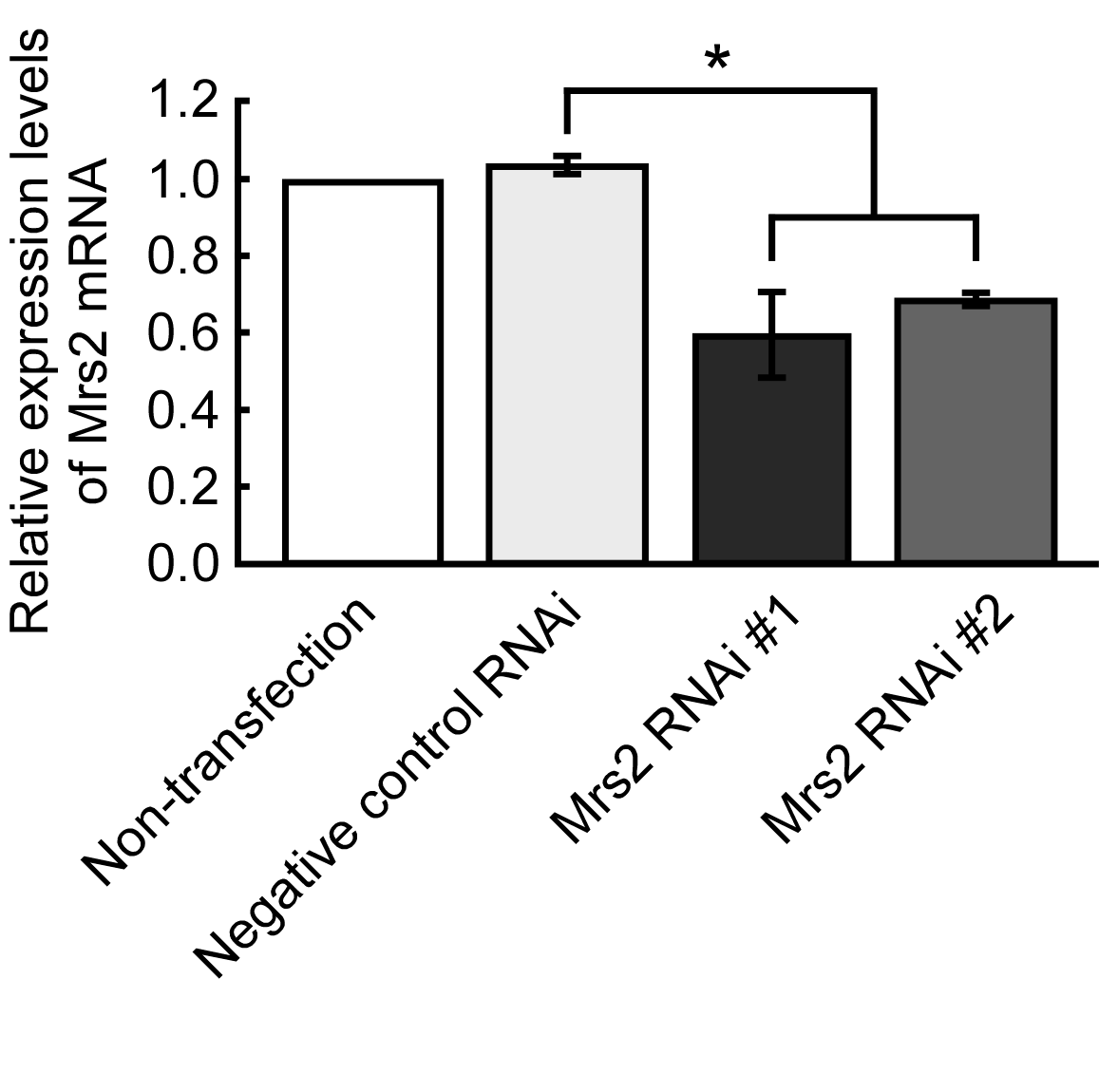

Supplement: Figure S3 — Knockdown of Mrs2 in PC12 cells. Relative expression levels of Mrs2 mRNA in PC12 cells transfected with expression vectors for miR RNAi against Mrs2 (Mrs2 RNAi #1 and #2), negative control miR RNAi and non–transfected cells were estimated with real-time PCR. In cells transfected with the miR RNAi against Mrs2, the expression levels of Mrs2 mRNA were suppressed. The efficiency of transfection of the vector to PC12 cells ranged from 40–50%. The efficiencies of suppression varied with the sequences of miR RNAi. Expression levels were measured in 3-4 different samples and compared with that in non-transfected cells. The error bars indicate SEM. * indicates p<0.05 estimated by using Tukey's test. (TIF) [file pone.0023684.s003.tif]

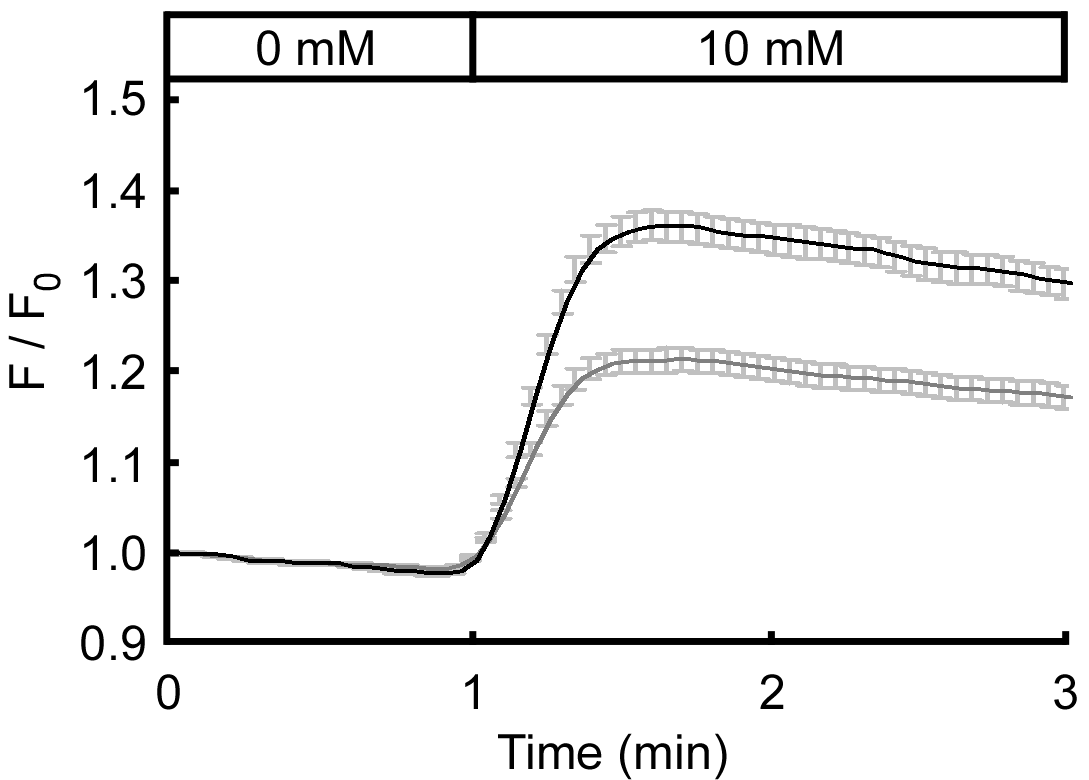

Supplement: Figure S4 — Mg2+ uptake into PC12 cells. The time-course of the change in [Mg2+]cyto (black line) and of the change in fluorescence of KMG-301 (gray line) was simultaneously measured in PC12 cells. Increasing the extracellular [Mg2+] from 0 mM to 10 mM induced a remarkable increase in [Mg2+]cyto and an increase in the fluorescence of KMG-301([Mg2+]mito). The error bars indicate SEM. (TIF) [file pone.0023684.s004.tif]

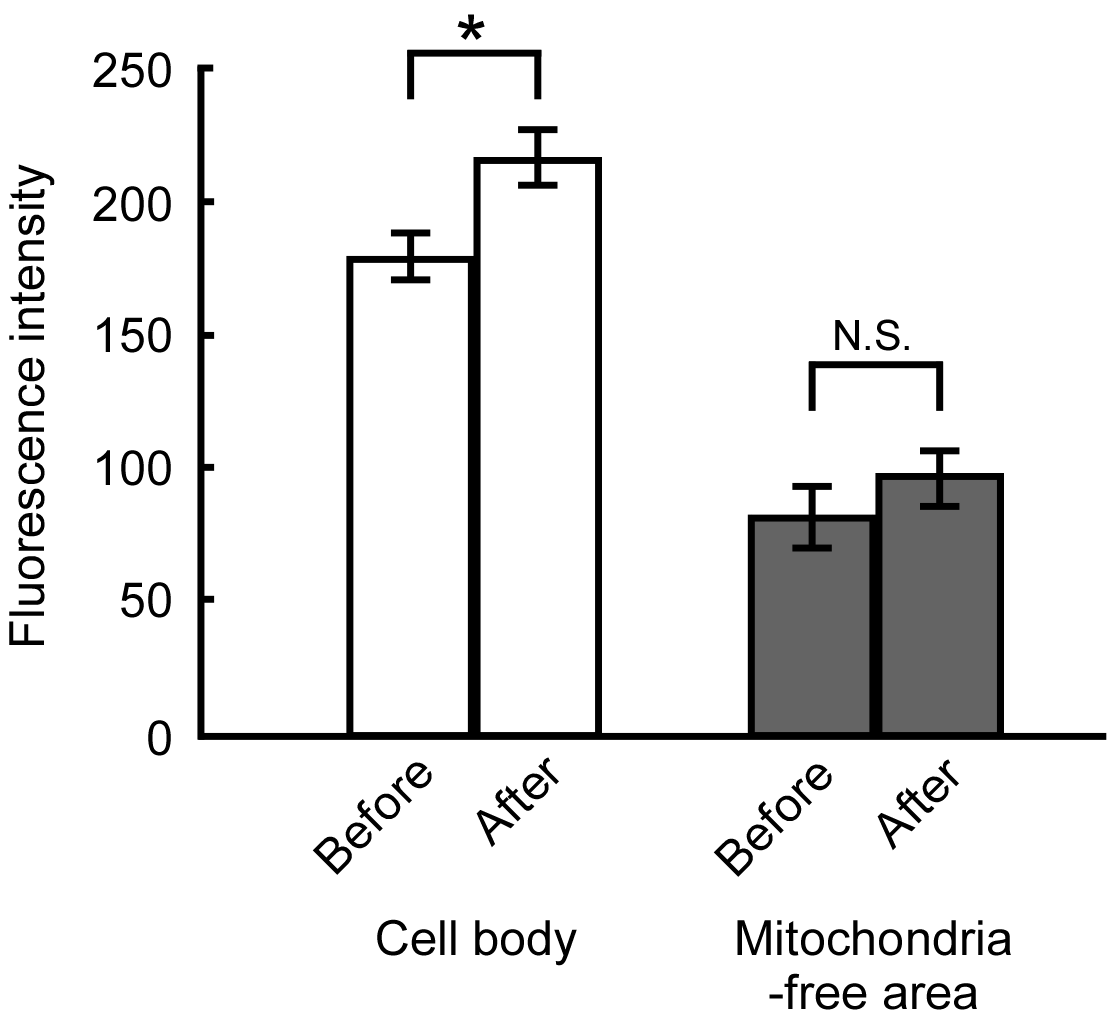

Supplement: Figure S5 — The fluorescence intensity of KMG-301 changed in the ROIs of the cell body. Non-differentiated PC12 cells expressing TagCFP-Mito (purchased from Evrogen, Moscow, Russia) were stained with KMG-301. As shown in Figure 4, an increase in extracellular Mg2+ concentration from 0 to 10 mM induced an increase in the fluorescence of KMG-301. Here, the fluorescence intensities of KMG-301 before and after application of 10 mM Mg2+ were compared in the ROI of the whole cell body, which contains mitochondria, and in the mitochondria-free area, which shows no fluorescence of TagCFP-Mito. Significant increases in the fluorescence intensity of KMG-301 were observed only in the ROIs of the whole cell body (N = 9, n = 20). The error bars indicate SEM. (TIF) [file pone.0023684.s005.tif]

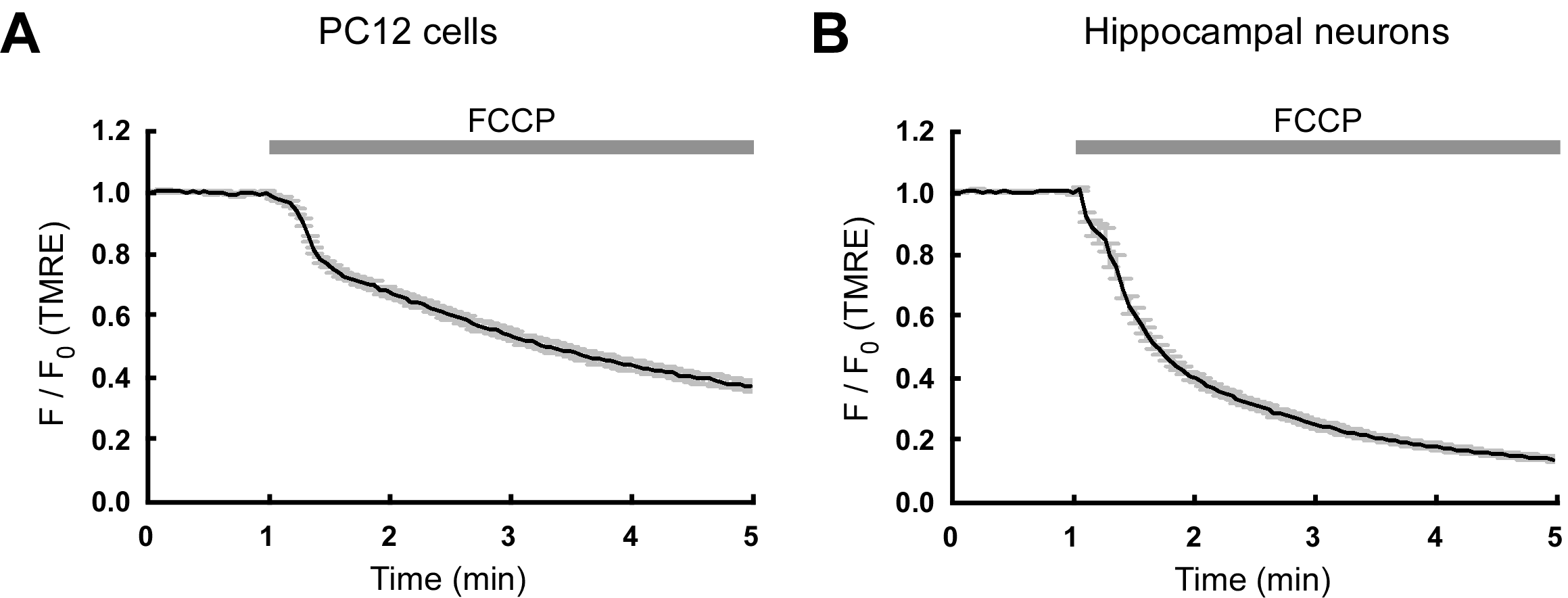

Supplement: Figure S6 — FCCP-induced depolarization of mitochondrial membrane potentials in differentiated PC12 cells and hippocampal neurons. (A) The time-course of FCCP-induced depolarization in PC12 cells was measured with TMRE. The mitochondrial membrane potential gradually decreased. (B) Time-course of FCCP-induced depolarization in hippocampal neurons. The error bars indicate SEM. (TIF) [file pone.0023684.s006.tif]

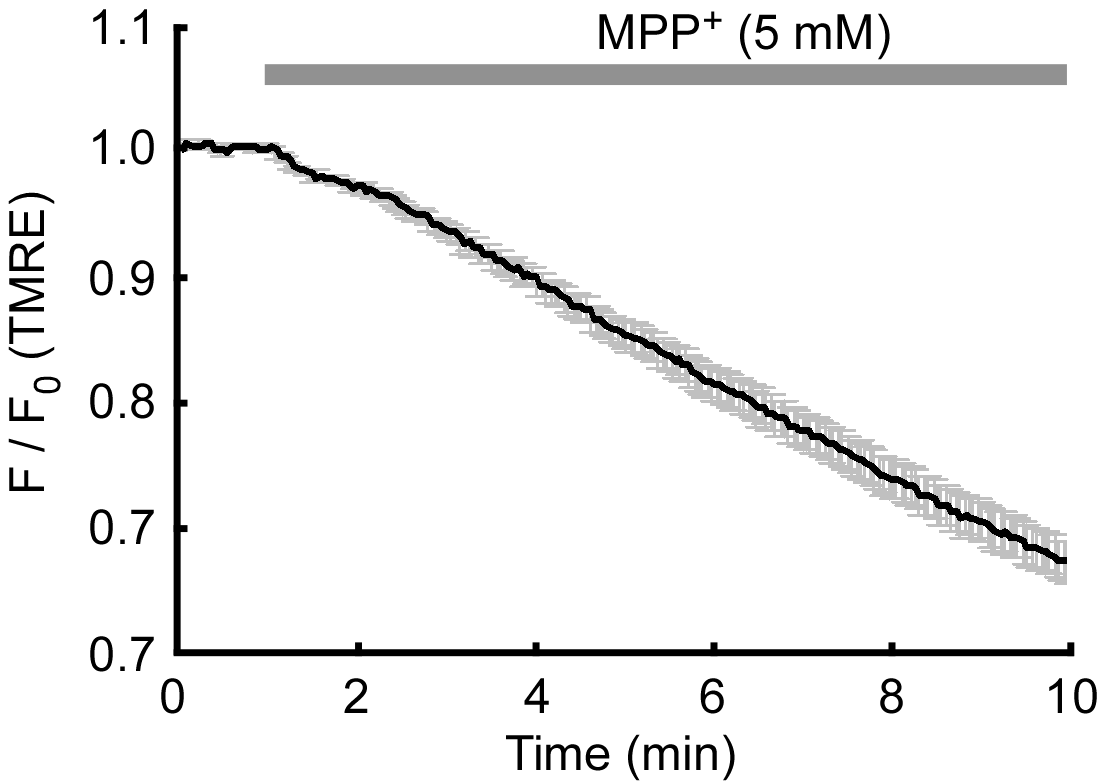

Supplement: Figure S7 — MPP+-induced depolarization of mitochondrial membrane potentials in differentiated PC12 cells. The time-course of MPP+-induced depolarization in differentiated PC12 cells was measured with TMRE. The mitochondrial membrane potential gradually decreased after MPP+ application. The error bars indicate SEM. (TIF) [file pone.0023684.s007.tif]
