# Supplementary material for: Newly Developed Mg2+–Selective Fluorescent Probe Enables Visualization of Mg2+ Dynamics in Mitochondria
Source: PLoS One. 2011 Aug 16;6(8):e23684. doi: 10.1371/journal.pone.0023684 (PMC3156752; doi:10.1371/journal.pone.0023684)
Supplement: Text S1 — The organic synthesis of KMG-301 and supporting methods are included in this file. (DOC) [file pone.0023684.s008.doc]

**Text S1**

**Synthesis of KMG-301 and KMG-301AM**

**Synthesis. General Procedures**

NMR spectra were obtained on JEOL JNM-EXC400, JNM-AL400 or JNM-ALPHA400 spectrometers [^1^H (400 MHz), ^13^C (100 MHz)] in deuterated methanol (CD_3_OH) solution using CD_2_HOD as an internal standard. High-resolution ESI-Mass spectra were obtained on a Waters LCT Premier XE. HPLC purification was carried out with a JASCO PU-2080 Plus pump equipped with a JASCO UVIDEC-100-V detector using a Senshu Pak PEGASIL ODS column (Senshu Scientific, Tokyo, Japan). The solvents used for HPLC were purchased from Nakalai Tesque (Tokyo, Japan) and filtered through a MILLIPORE OMNIPORE^TM^ membrane filter (0.45 μm pore size, 47 mm. diameter; Millipore, Billerica, MA, USA) before use. Silica gel column chromatography was performed on silica gel 60N (spherical, neutral, 63-210 μm, Kanto Chemical, Tokyo, Japan). Reversed-phase open-column chromatography was performed on PEGASIL PREP ODS-7515-12A (Senshu Scientific). Thin layer chromatography (TLC) was performed on silica gel plates F_254_ (0.25 mm (analytical) and 0.50 mm (preparative), MERCK, NJ, USA).

**Synthesis of KMG-301 and KMG-301AM. (a) Ethyl 1-(bis(4-(dimethylamino)-2- hydroxyphenyl)methyl)-4-oxo-4*H*-quinolizine-3-carboxylate (4).**

To a stirred solution of ethyl 1-formyl-4-oxo-4*H*-quinolizine-3-carboxylate (1) (**3**: 52 mg, 0.21 mmol) and 3-(dimethylamino)phenol (70 mg, 0.51 mmol) in acetic acid (2.0 mL) was added *p*-toluenesulfonic acid monohydrate (5 mg, catalytic amount) at room temperature under Ar, and the reaction mixture was warmed to 60°C. After being stirred at the same temperature for 24 hours, the solvent was removed *in vacuo*, and sat. NaHCO_3_ aq. (20 mL) was added to the residue, and extracted with chloroform 3 times. The combined extracts were washed with brine, dried with Na_2_SO_4_, and evaporated. The residue was purified by silica gel column chromatography (chloroform-methanol = 15:1) to give 85 mg (80%) of **4** as a dark yellow powder.

**4**: ^1^H-NMR (400 MHz, CD_3_OH, rt): 9.48 (1H, d, *J* = 7.2 Hz), 7.99 (1H, d, *J* = 8.9 Hz), 7.94 (1H, s), 7.74 (1H, t, *J* = 6.8 Hz), 7.38 (1H, t, *J* = 6.8 Hz), 6.60 (2H, d, *J* = 8.6 Hz), 6.31 (2H, d, *J* = 2.5 Hz), 6.21 (2H, dd, *J* = 2.5, 8.6 Hz), 6.21 (1H, s), 4.22 (2H, q, *J* = 7.3 Hz), 2.78 (12H, s), 1.25 (3H, t, *J* = 7.3 Hz) ppm.; ^13^C-NMR (100 MHz, CD_3_OH, rt): 167.7, 156.9, 148.6, 146.1,140.8, 136.0, 131.7, 130.9, 124.1, 118.8, 107.8, 104.8, 103.9, 103.8, 61.7, 43.2, 38.4, 14.6 ppm.; HR ESI MS (positive): [M^+^H]^+^ Found *m/z* 502.2333, C_29_H_32_N_3_O_5_ requires *m/z* 502.2342.

**(b) *N*-(6-(dimethylamino)-9-(3-(ethoxycarbonyl)-4-oxo-4*H*-quinolizin-1-yl)-3*H*-xanthen- 3-ylidene)-*N*-dimethylammonium trifluoroacetate (5, KMG-301 ethyl ester).**

To a stirred solution of **4** (25 mg, 0.049 mmol) in acetic acid-benzene 1:1 mixture (1.5 mL) was added DDQ (23 mg, 0.098 mmol) at room temperature under Ar. The color of the reaction mixture changed quickly from clear yellow to dark purple. After stirring for 2 hours, the solvent was removed *in vacu*o and the residue was purified by silica gel column chromatography (chloroform-methanol = 5:1; 0.1% TFA was then added to elute). Furthermore the crude product was purified by reversed-phase open-column chromatography (60% MeOH) to give 21 mg (89%) of **5** as a purple compound.

**5**: ^1^H-NMR (400 MHz, CD_3_OD, rt): 9.62 (1H, d, *J* = 6.8 Hz), 8.42 (1H, s), 7.86 (1H, t, *J* = 7.8 Hz), 7.60 (1H, t, *J* = 6.8 Hz), 7.49 (1H, d, *J* = 8.8 Hz), 7.40 (2H, d, *J* = 9.3 Hz), 7.07 (2H, dd, *J* = 2.5, 9.3 Hz), 7.01 (2H, d, *J* = 2.5 Hz), 4.40 (2H, q, *J* = 6.8, Hz), 3.42 (12H, s), 1.38 (3H, t, *J* = 6.8 Hz) ppm.; ^13^C-NMR (100 MHz, CD_3_OD, rt) 159.5, 159.2, 159.0, 142.2, 132.0, 131.7, 120.8, 116.0, 97.8, 46.8, 41.0, 14.4 ppm.; HR ESI MS (positive) : [M]^+^ Found *m/z* 482.2070, C_29_H_28_N_3_O_4_ requires *m/z* 482.2080.

**(c) *N*-(9-(3-carboxy-4-oxo-4*H*-quinolizin-1-yl)-6-(dimethylamino)-3*H*-xanthen-3-ylidene)- *N*-methylmethanaminium trifluoroacetate (1, KMG-301).**

To a stirred solution of **5** (21 mg, 0.042 mmol) in methanol (1.0 mL) was added KOH aq. (2.0 M, 1.0 mL) at 0 °C and the reaction mixture was warmed to room temperature. After being stirred for 2 hours, 1N HCl was added into the reaction mixture and the pH was adjusted to 3-4, and the solvent was removed *in vacuo*. The residue was purified by reversed-phase open-column chromatography (washed with 40% MeOH and eluted with 100% MeOH) to give 20 mg (quant.) of **1** as a dark red compound.

**1**: ^1^H-NMR (400 MHz, CD_3_OD, rt): 9.60 (1H, d, *J* = 4.5 Hz), 8.45 (1H, s), 7.91 (1H, t, *J* = 6.7 Hz), 7.68 (1H, t, *J* = 6.7 Hz), 7.57 (1H, d, *J* = 8.3 Hz), 7.40 (2H, d, *J* = 9.3 Hz), 7.06 (2H, d, *J* = 9.3 Hz), 7.02 (2H, s), 3.33 (12H, s) ppm.; ^13^C-NMR (100 MHz, CD_3_OD, rt) 159.4, 159.2, 138.8, 138.7, 132.1, 131.2, 125.4, 120.9, 120.8, 116.0, 115.9, 97.8, 41.1 ppm.; HR ESI MS (positive) : [M]^+^ Found *m/z* 454.1757, C_27_H_24_N_3_O_4_ requires *m/z* 454.1767.

**(d) *N*-(9-(3-((acetoxymethoxy)carbonyl)-4-oxo-4*H*-quinolizin-1-yl)-6-(dimethylamino)- 3*H*-xanthen-3-ylidene)-*N*-methylmethanaminium trifluoroacetate (2, KMG-301AM).**

To a stirred solution of **1** (14 mg, 0.029 mmol) in acetonitrile-methanol 4:1 mixture (1.0 mL) was added triethylamine (50 μL, 0.36 mmol) and bromomethyl acetate (15 μL, 0.15 mmol) at room temperature under Ar. After being stirred for 4 hours, an additional 15 μL of bromomethyl acetate was added to the reaction mixture, and stirring was continued for 15 hours. The solvent was removed *in vacuo*, and the residue was purified twice by silica gel chromatography (at first, chloroform-methanol = 5:1 containing 0.1% TFA, and second, 80% acetonitrile). Further purification of the crude product was performed with HPLC [Senshu Pak PEGASIL ODS (10φ x 150 mm), 40% MeOH] to give 11 mg (69%) of **2** as a red compound.

**2**: ^1^H-NMR (400 MHz, CD_3_OD, rt): 9.61 (1H, d, *J* = 7.3 Hz), 8.38 (1H, s), 7.85 (1H, t, *J* = 7.3 Hz), 7.59 (1H, t, *J* = 7.3 Hz), 7.48 (1H, d, *J* = 7.3 Hz), 7.45 (2H, d, *J* = 9.3 Hz), 7.08 (2H, dd, *J* = 2.4, 9.3 Hz), 7.04 (2H, d, *J* = 2.4 Hz), 5.94 (2H, s), 3.35 (12H, s), 2.08 (3H, s) ppm.; ^13^C-NMR (100 MHz, CD_3_OD, rt) 171.3, 159.5, 159.2, 146.7, 142.4, 139.4, 132.1, 131.8, 125.3, 120.6, 116.0, 97.8, 81.1, 41.1, 20.6 ppm.; HR ESI MS (positive) : [M]^+^ Found *m/z* 526.1982, C_30_H_28_N_3_O_6_ requires *m/z* 526.1978.

**NMR spectra of compounds**

**
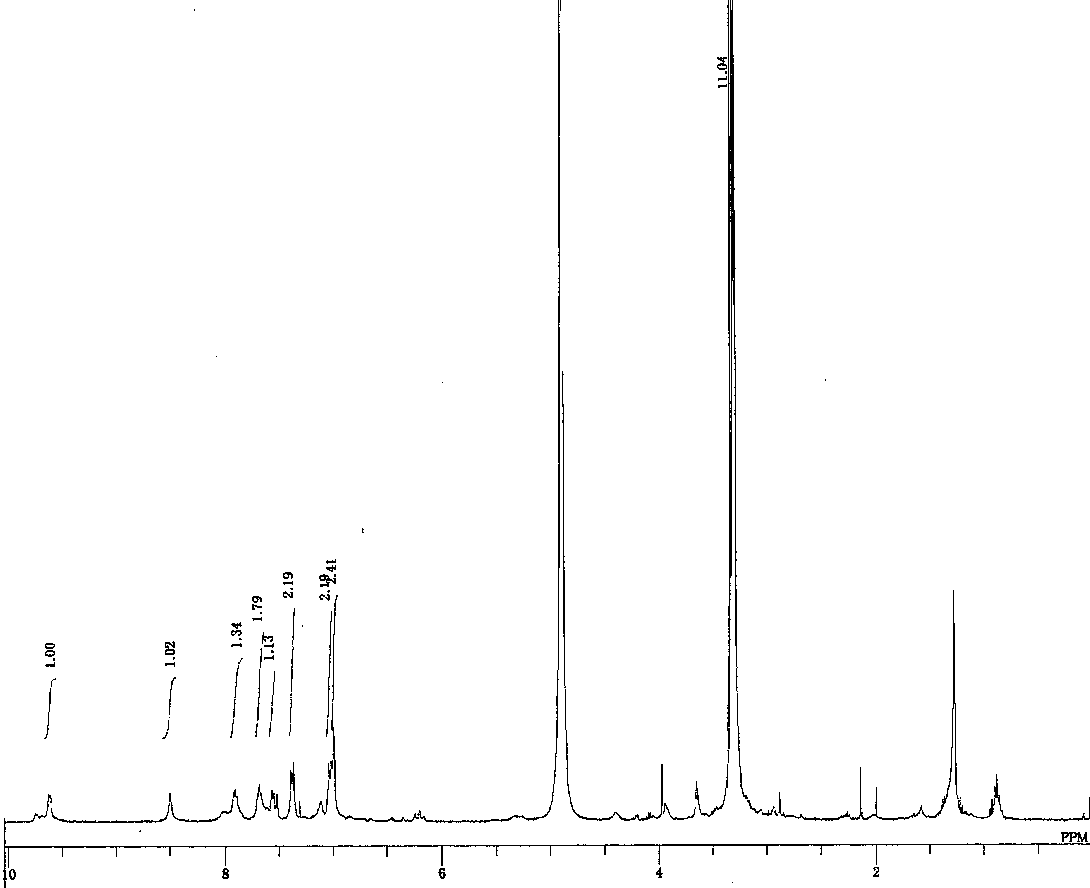
**

Compound **4**, ^1^H-NMR


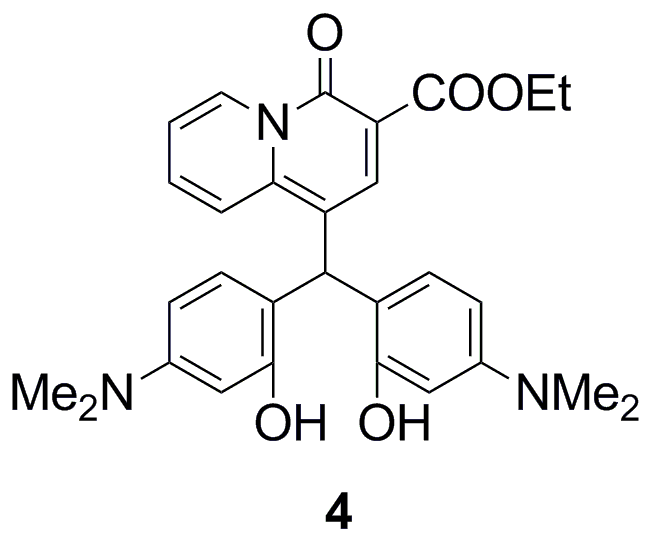


**4**, ^13^C-NMR


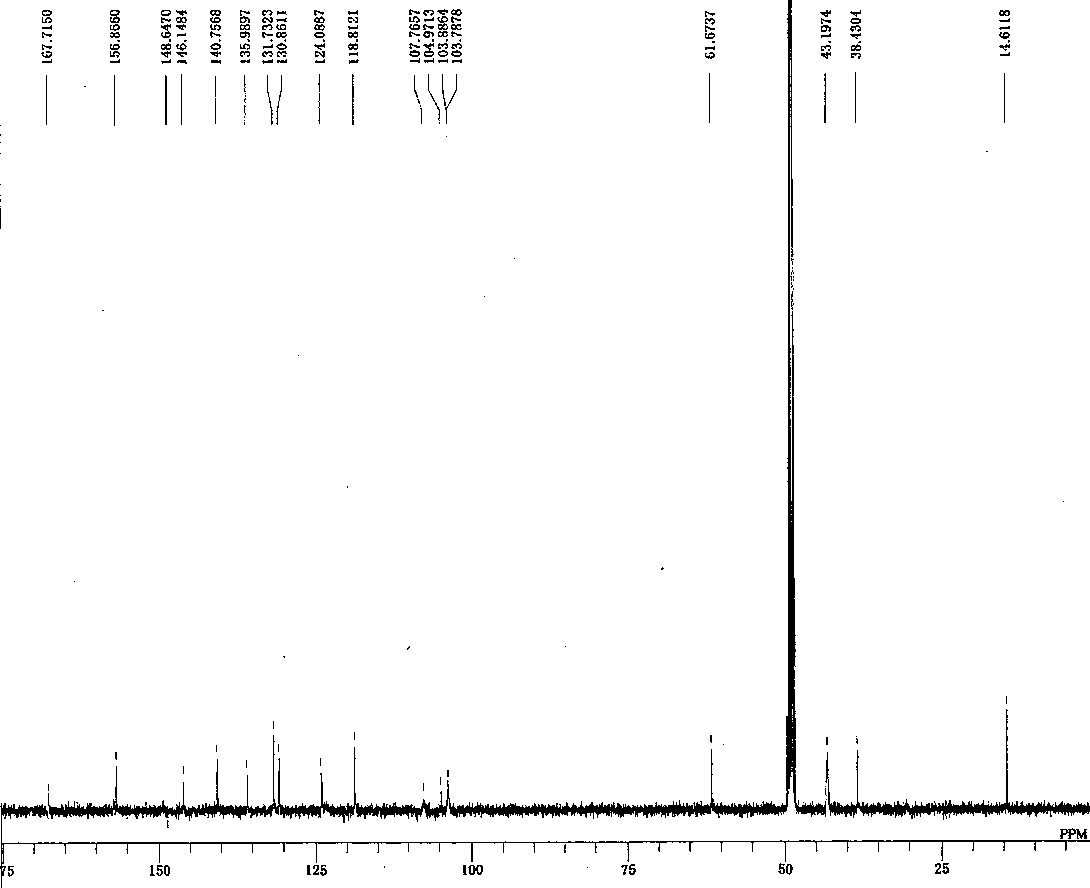


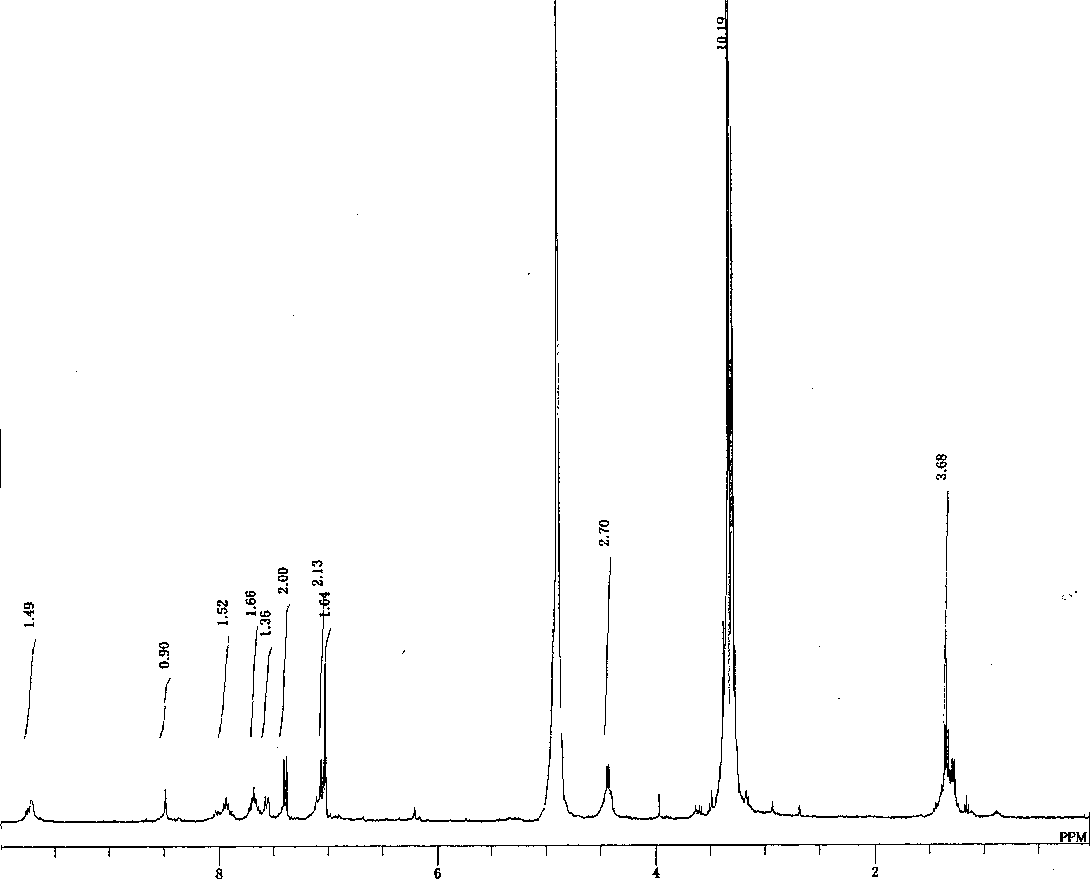


Compound **5**, ^1^H-NMR


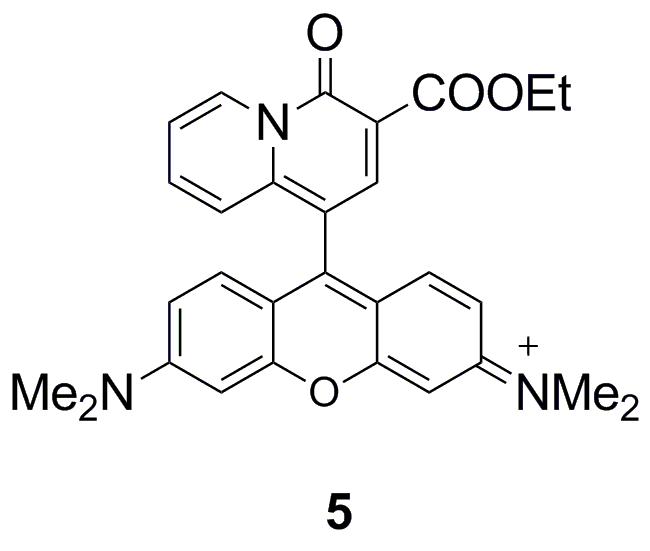


**5**, ^13^C-NMR


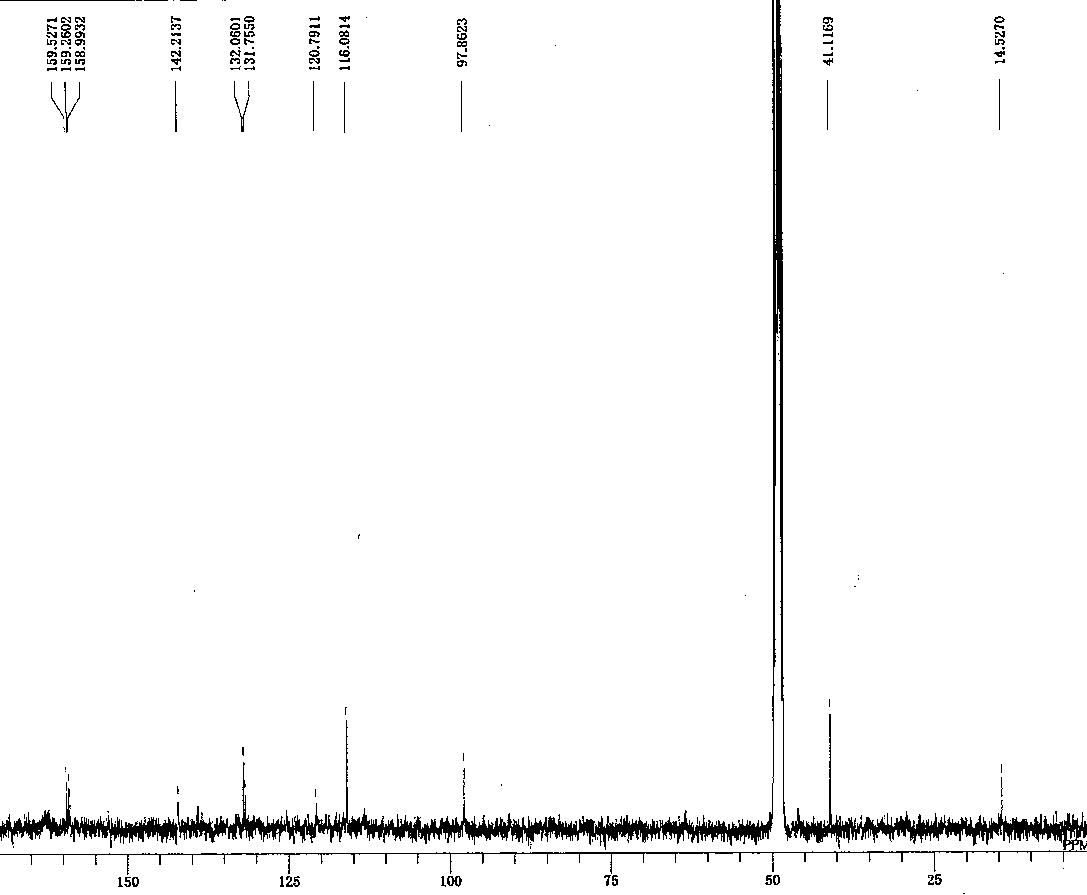


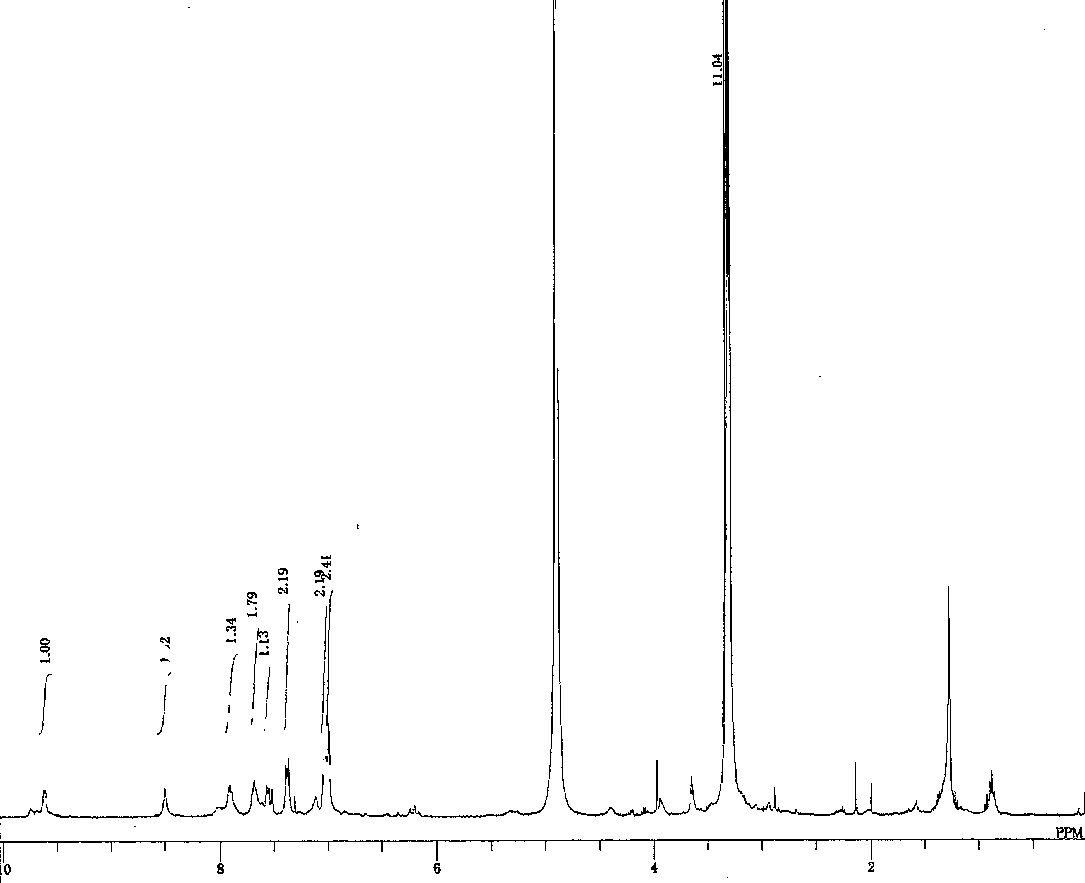


Compound **1**, ^1^H-NMR


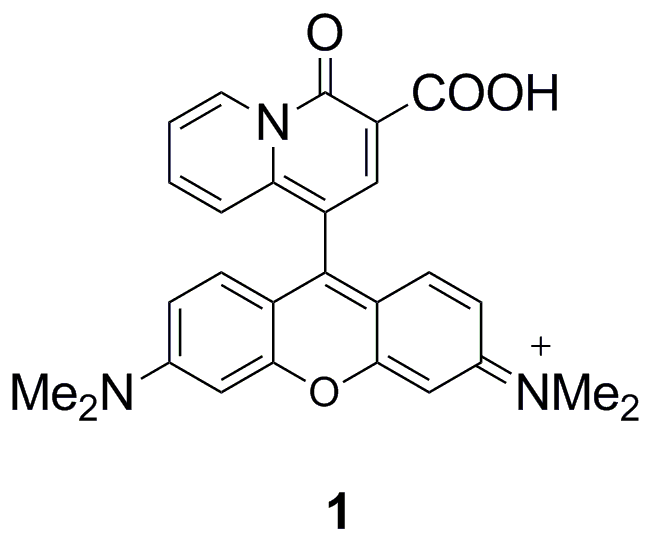


**1**, ^13^C-NMR


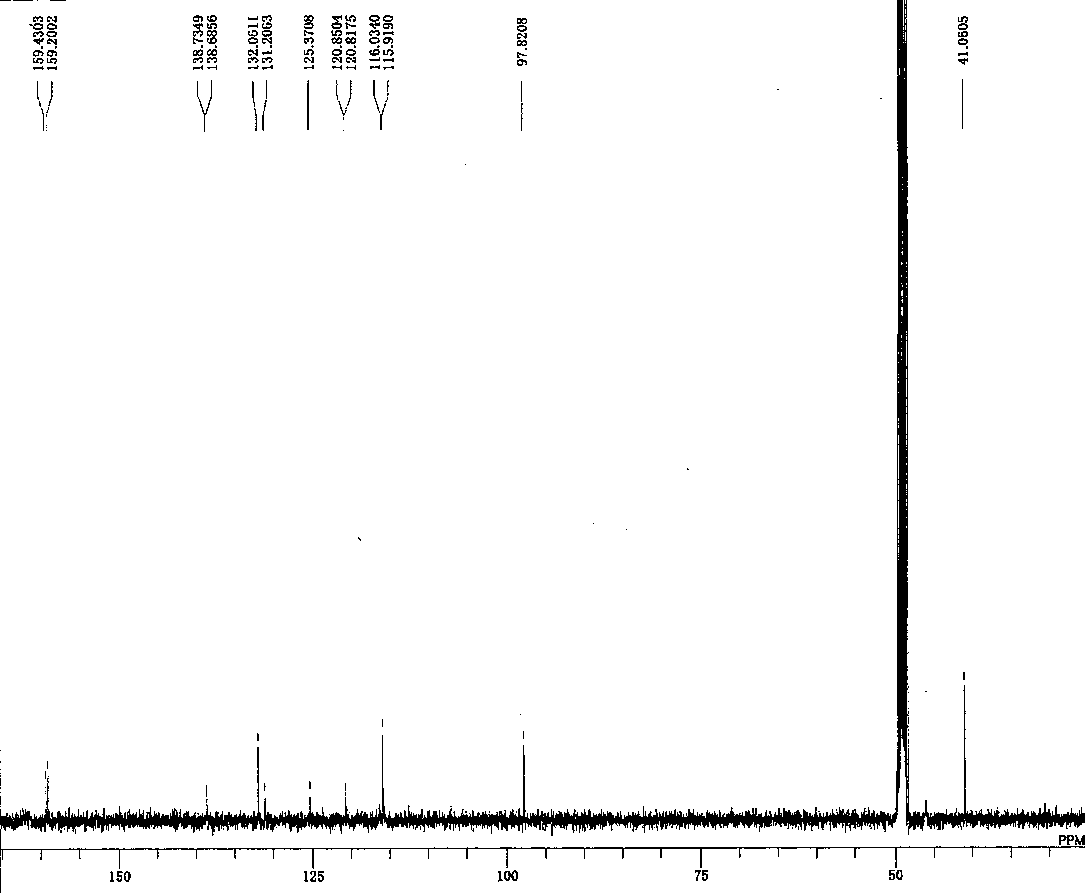


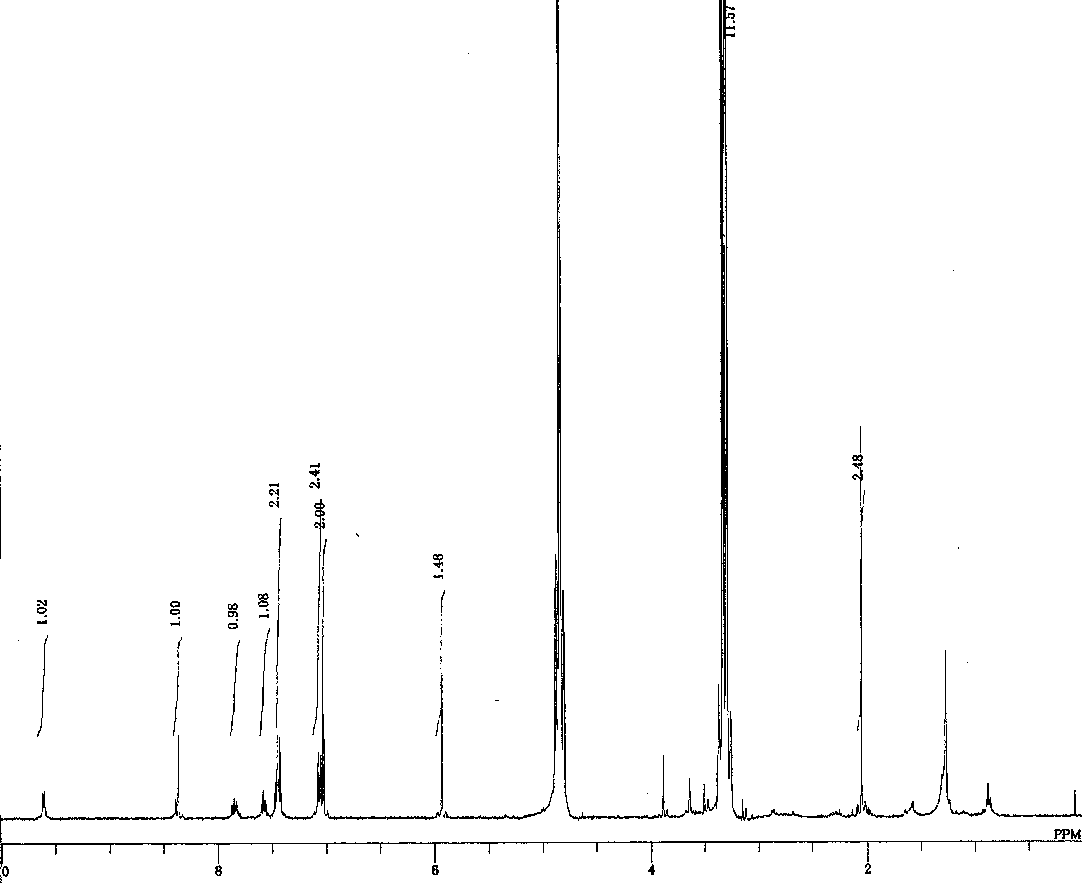


Compound **2**, ^1^H-NMR


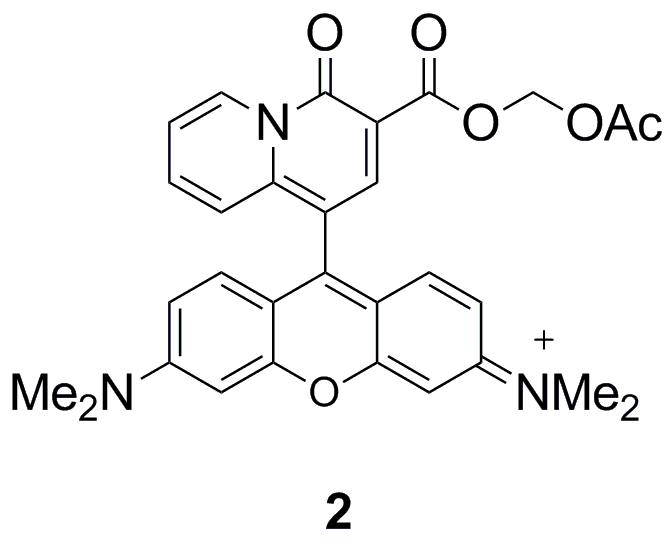


**2**, ^13^C-NMR


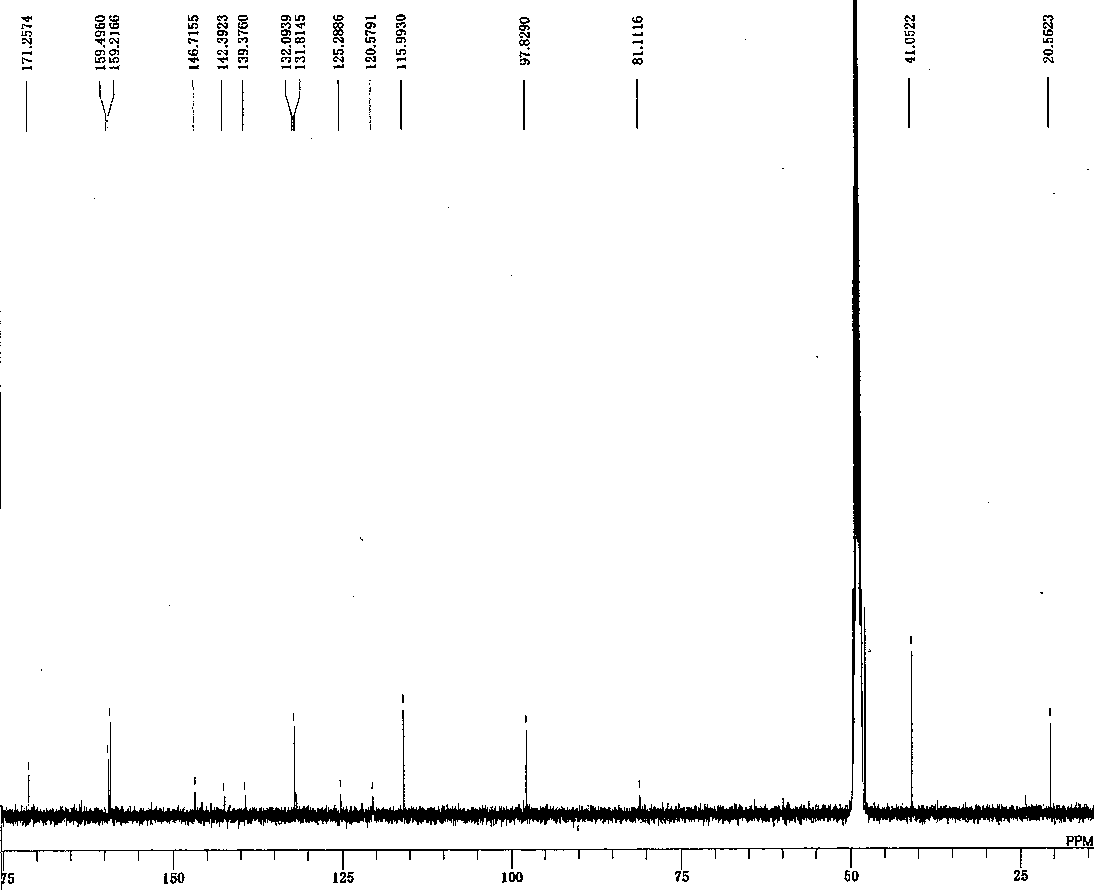


**Knock down of Mrs2 protein**

miR RNAi-mediated knockdown was performed using BLOCK-iT^TM^ PolII miR RNAi Expression Vector Kit with EmGFP (Invitrogen, Carlsbad, CA, USA). The miR RNAis used were as follows:

Mrs2 #1,

5’-TGCTGTAAACCGGTACACATCACCTGGTTTTGGCCACTGACTGACCAGGTGATGTACCGGTTTA-3’

Mrs2 #2,

5’-TGCTGTTCACATCCATCCTTCTATCTGTTTTGGCCACTGACTGACAGATAGAAATGGATGTGAA-3’

These oligos were cloned into the pcDNA^TM^6.2-GW/EmGFP-miR expression vector.

The negative control plasmid contains an insert that can form a hairpin structure that is processed into mature miRNA, but is predicted not to target any known vertebrate gene. The negative control sequence is shown below:

5’-GAAATGTACTGCGCGTGGAGACGTTTTGGCCACTGACTGACGTCTCCACGCAGTACATTT-3’

The expression vector was transfected to PC12 cells by electroporation using Neon (Invitrogen). Experiments were performed 4-days after transfection. The expression of miR RNAi was confirmed by the co-expression of emerald GFP (EmGFP). In the fluorescence measurements, the fluorescence of KMG-301 was observed only in EmGFP-expressing cells.

**Real-time PCR**

Total RNA from PC12 cells was isolated and purified by using the RNeasy mini kit (QIAGEN, Tokyo, Japan). The total RNA was treated with Terbo DNase (Ambion, CA, USA), and Single-stranded cDNA was generated by reverse transcription by using SuperScript VILO (Invitrogen).

Real-time PCR was carried out with SYBR GreenER^TM^ (Invitrogen). Primers used were as follows: for Mrs2 (forward primer, 5’- AGCATCACTACCAGAAACAACAGG; reverse primer, 5’- GGAACAGCCAGTGCTCCAA) and for GAPDH (forward primer, 5’- GATGGGTGTGAACCACGAGA; reverse primer, 5’- AGTGATGGCATGGACTGTGG). Real-time PCR was performed by using Rotor-Gene (QIAGEN) for 40 cycles. Data was quantified with the comparative Ct quantification method by plotting cycle number at threshold and expressed in arbitrary units relative to the level of the same gene expression in cells expressing negative control miR RNAi, relative to GAPDH.

**Reference**

1. Otten, P. A., London, R. E., and Levy, L. A. (2001) 4-oxo-4H-quinolizine-3-carboxylic acids as ^Mg^2+ selective, fluorescent indicators*, Bioconjug. Che*m*.* 12, 203-212.
